# Dissecting Immune Determinants in Lesional Skin of Cutaneous T-Cell Lymphoma During Mogamulizumab Therapy

**DOI:** 10.3390/cancers18142348

**Published:** 2026-07-21

**Authors:** Xiao Ni, Wei Han, Niharika Kunta, Meghali Goswami, Jared K. Burks, Ye Zheng, Youn H. Kim, Madeleine Duvic

**Affiliations:** 1 Department of Dermatology, The University of Texas MD Anderson Cancer Center, Houston, TX 77030, USA; whan3@mdanderson.org (W.H.); niharika.kunta@bcm.edu (N.K.); mduvic2020@outlook.com (M.D.); 2 Department of Hematopoietic Biology and Malignancy, The University of Texas MD Anderson Cancer Center, Houston, TX 77030, USA; jburks@mdanderson.org; 3 Department of Leukemia, The University of Texas MD Anderson Cancer Center, Houston, TX 77030, USA; 4 Department of Systems Biology, The University of Texas MD Anderson Cancer Center, Houston, TX 77030, USA; yzheng8@mdanderson.org; 5 Department of Bioinformatics and Computational Biology, The University of Texas MD Anderson Cancer Center, Houston, TX 77030, USA; 6 Department of Dermatology, Stanford University School of Medicine, Stanford, CA 94305, USA; younkim@stanford.edu

**Keywords:** cutaneous T-cell lymphoma (CTCL), mycosis fungoides, Sézary syndrome, CCR4, mogamulizumab, tumor microenvironment, imaging mass cytometry

## Abstract

**Background**: Mycosis fungoides (MF) and Sézary syndrome (SS) are the predominant cutaneous T-cell lymphomas. Although malignant T cells in MF/SS overexpress CCR4 and respond to the anti-CCR4 antibody mogamulizumab, skin response rates vary. We hypothesized that immune components within the tumor microenvironment contribute to differential outcomes. **Methods**: Imaging mass cytometry with a 37-antibody panel was used to characterize immune and structural elements in FFPE tissues from sixteen MF/SS patients (6 MF, 10 SS) treated with Mogamulizumab, including seven skin responders and nine non-responders. Single-cell phenotyping and spatial analyses were performed using the Visiopharm^®^ Phenoplex™ platform, with supervision. **Results**: We identified 68,974 cells pre-treatment and 58,852 cells post-treatment. Malignant CD4^+^ T cells showed reduced baseline CD27, CD103, CD25, and ICOS expression compared with non-malignant CD4^+^ cells. Baseline MF lesions were enriched for IL-13^+^ and CD103^+^ malignant T cells, whereas SS lesions contained higher proportions of CD27^+^ and LAG3^+^ cells. IL 13^+^ malignant cells decreased after treatment, most prominently in MF. Myeloid profiles differed by disease and response: MF lesions exhibited baseline enrichment of M1-like macrophages (CD86^+^, HLA-DR^+^), while SS lesions were predominantly M2-polarized macrophages (CD163^+^, CD206^+^). Responders showed increased M1-like macrophages, whereas non-responders displayed reduced M1-features. An increase in DC3-like cells was observed in non-responders following treatment. **Conclusions**: This single-cell spatial atlas reveals shared and subtype-specific immune features in MF/SS. Th2-skewed malignant T-cell status and myeloid polarization correlate with clinical response, supporting their potential as spatial biomarkers for patient stratification in mogamulizumab therapy.

## Simple Summary

Mycosis fungoides (MF) and Sézary syndrome (SS) are the predominant cutaneous T-cell lymphoma, but patient responses to the anti-CCR4 monoclonal antibody mogamulizumab are highly variable and remain difficult to predict. To identify tissue features associated with therapeutic response, we applied imaging mass cytometry to comprehensively map malignant and non-malignant immune cell populations within lesional skin from MF and SS patients. Our analyses revealed distinct subtype-specific immune landscapes, including differences in T-cell phenotypes and myeloid cell programs associated with treatment outcomes. These findings provide new insights into the tumor microenvironment and support the development of predictive biomarkers and improved patient stratification strategies for mogamulizumab therapy.

## 1. Introduction

Mycosis fungoides (MF) and Sézary syndrome (SS) are the predominant cutaneous T-cell lymphomas (CTCLs), characterized by high symptom burden, chronic relapsing courses, and limited curative options. Although comprehensive genomic, transcriptomic, and proteomic profiling has advanced molecular understanding, the cutaneous tumor microenvironment (TME), including the immune and stromal population composition, functional states, and spatial organization, remains comparatively underexplored in MF/SS [1,2]. This gap is clinically relevant because skin responses to systemic therapies often diverge from blood responses, suggesting skin-specific determinants of sensitivity and resistance.

CC chemokine receptor 4 (CCR4) is frequently overexpressed on malignant T cells in CTCL, providing a rational target for immunotherapy. Mogamulizumab, a humanized anti-CCR4 monoclonal antibody, mediates antibody-dependent cellular cytotoxicity (ADCC) against CCR4^+^ malignant T cells and CCR4^+^ regulatory T cells (Tregs) and has been FDA-approved for MF/SS [3,4]. Nevertheless, clinical benefit is modest overall, with an overall response rate of 36.8% (47.1% in SS; 28.6% in MF) and generally inferior responses in skin (42.1%) compared with blood (94.7%) [4]. These observations imply that CCR4 expression on malignant cells, while necessary for target engagement, is insufficient to fully predict or drive clinical response in the cutaneous compartment.

Prior correlative analyses demonstrated consistent depletion of CCR4^+^ T cells in lesional skin after mogamulizumab treatment irrespective of clinical outcome, confirming on-target activity while implicating TME features beyond CCR4 expression as modulators of efficacy [3]. Historically, immune profiling in CTCL has emphasized peripheral blood by flow cytometry, whereas tissue studies have often been limited to low-plex immunohistochemistry (e.g., CCR4 and FOXP3), leaving skin-resident cellular ecosystems and their spatial inter-actions insufficiently resolved.

To address these gaps, we applied imaging mass cytometry (IMC)—a time-of-flight mass cytometry platform that enables highly multiplexed, spatially resolved, single-cell phenotyping in situ—to quantify cellular compositions, cell states, neighborhood relationships, and context-specific immune circuits within MF/SS lesional skin during mogamulizumab therapy [5,6]. This approach allows simultaneous visualization of malignant and non-malignant T-cell programs, myeloid polarization states, and suppressive or effector niches that may shape response or resistance.

Our single-cell spatial atlas reveals that Th2-skewed malignant T cells and myeloid polarization are associated with skin response, supporting their potential as spatial biomarkers for patient stratification in mogamulizumab treatment.

## 2. Materials and Methods

### 2.1. Patient Cohort

The cohort of 16 patients was drawn from a previously published study [1] based on the availability of archived formalin-fixed, paraffin-embedded (FFPE) tissue blocks and/or precut FFPE sections [3,4]. The MD Anderson Cancer Center Institutional Review Board (PA14-0177) approved the study. This cohort comprised 6 patients with mycosis fungoides (MF) and 10 with Sézary syndrome (SS) (Table S1). Fifteen patients received mogamulizumab at 1.0 mg/kg, and one patient (0303) received 0.3 mg/kg. Clinical responses to mogamulizumab were evaluated according to established consensus criteria, with the modified Severity-Weighted Assessment Tool (mSWAT) used for assessment of skin disease burden in all patients. Three patients (108, 109, 310) achieved a partial skin response after one course (3/16, 18.8%), and four patients (117, 307, 303, 319) demonstrated skin responses after Course 3 or Course 5, for a total of seven skin responders (7/16, 43.8%). Paired samples were collected pre-treatment or Day 1 and after one cycle of treatment or Day 29, as reported in our previous study [3].

### 2.2. Tissue Specimens, H&E Staining and Conventional and Dual-Color Immunohistochemistry

Serial 5 µm sections were cut from paired FFPE blocks for seven patients from MD Anderson Cancer Center. Paired precut FFPE sections for nine patients from Stanford University School of Medicine were retrieved. Hematoxylin and eosin (H&E) staining was used for routine histopathology and selection of regions of interest (ROIs). Conventional IHC was performed to validate individual antibodies and assess staining patterns. Dual-color IHC for CCR4 and CD1a was performed on selected sections using the ImmPRESS^®^ Duet Double Staining HRP/AP Polymer Kit (Vector Laboratories, Newark, CA, USA, MP-7714-15), according to the manufacturer’s instructions.

In brief, 5-µm sections were baked at 60 °C for 4 h, deparaffinized in xylene, and re-hydrated through a graded ethanol series. Antigen retrieval was carried out in Tris-based Antigen Unmasking Solution (pH 9.0, Vector Laboratories, Newwark, CA, USA) for 30 min. Endogenous peroxidase activity was quenched with BLOX-ALL^®^ Blocking Solution (Vector Laboratories, Newwark, CA, USA) for 10 min, followed by a 10 min wash in Tris-buffered saline (TBS) containing 0.1% Triton X-100. Sections were then blocked with 2.5% normal horse serum for 20 min. Primary antibodies were applied overnight at 4 °C: rabbit polyclonal anti-human CCR4 (1:250; Novus Biologicals, Centennial, CO, USA, NBP1-86584) and mouse monoclonal anti-human CD1a (clone O10; 1:1200; Novus Biologicals, Centennial, CO, USA, NBP2-34697). Antibodies were diluted in TBST (TBS + 0.05% Tween-20) containing 2% bovine serum albumin (BSA). After buffer washes, sections were incubated with the ImmPRESS^®^ Duet reagent (Vector Laboratories, Newwark, CA, USA) for 10 min. Chromogenic development was performed sequentially with ImmPACT^®^ DAB EqV (Vector Laboratories, Newwark, CA, USA) (2–10 min) followed by ImmPACT^®^ Vector^®^ Red (Vector Laboratories, Newwark, CA, USA) (20–30 min), with a buffer wash between chromogens. Slides were counterstained with hematoxylin.

### 2.3. Antibody Panel and Metal Conjugation for Imaging Mass Cytometry

A 37-antibody panel was assembled to profile immune and non-immune components of the cutaneous tissue microenvironment by imaging mass cytometry (IMC). Each antibody was conjugated to a unique heavy-metal isotope (Table S2). Two thirds of the antibodies were pre-conjugated and obtained from commercial suppliers or from the Flow Cytometry and Cellular Imaging Core Facilities at The University of Texas MD Anderson Cancer Center and Baylor College of Medicine (Houston, TX, USA). One third of anti-bodies were conjugated in-house using the Maxpar^®^ Antibody Labeling Kit (Standard BioTools, Boston, MA, USA) according to the manufacturer’s instructions. Briefly, prior to conjugation, antibodies were validated by conventional IHC on FFPE tonsil sections. Assay conditions for individual markers were optimized by single-marker staining. Antibodies that showed high tissue background, inappropriate cellular localization, or failed to produce the expected expression pattern after segmentation-based marker quantification (i.e., CD1a, CCR4, CD56, CD123, CD69, CD141, and IFNG) were excluded from downstream analyses, leaving 30 markers in the final list. All metal-conjugated antibodies were stored at 0.5 mg/mL in phosphate-buffered saline (PBS) with stabilizer at 4 °C. Full details of antibody clone, supplier, catalog number, metal isotope tag, and working dilution) are provided in supplemental Table S2.

### 2.4. Imaging Mass Cytometry Staining

IMC staining was performed according to methods described by Ijsselsteijn et al. with modifications [5]. In brief, paired pre- and post-treatment tissue sections from the same patients were mounted on the same slide (Superfrost^®^ Plus, Thermo Fisher Scientific, Waltham, MA, USA). Each slide accommodated 3–4 pairs of sections plus 1–2 control sections. Slides were baked at 60 °C for 2 h, then deparaffinized in fresh xylene and rehydrated through a graded alcohol series (100%, 96%, 70%; 5 min each). Heat-induced epitope retrieval was performed in Tris–EDTA (pH 9.2) buffer at 95–98 °C for 30 min in a water bath. After cooling to room temperature, sections were blocked with 2% horse serum and 1% BSA for 1.5 h and incubated with the metal-isotope-tagged antibody cocktail for either 5 h at room temperature or overnight at 4 °C (as indicated in Table S2) in a humidified chamber. Following washes in TBST and TBS, nuclei were labeled with a freshly prepared Iridium (Ir) 191/193 DNA intercalator (Standard BioTools, Boston, MA, USA) to enable identification and segmentation of individual cells. Slides were then washed in TBS, rinsed briefly in water, and air-dried prior to IMC image acquisition.

### 2.5. IMC Image Acquisition

Imaging mass cytometry was performed on the Hyperion™ XTi Imaging System (Standard BioTools, Boston, MA, USA) at a laser repetition rate of 200 Hz by the Flow Cytometry and Cellular Imaging Core Facility at The University of Texas MD Anderson Cancer Center (Houston, TX, USA). For each sample, regions of interest (ROIs) were selected from corresponding H&E sections to capture dense infiltrates. Preview Mode on the Hyperion Imaging System was performed on five paired tissue sections to guide ROI selection (Figure S1; E-cadherin: red, CD45: green, collagen I: blue). Cell Mode was used to analyze single-cell spatial marker expression for targeted ROIs across all tissue sections. Following ablation, raw MCD files were inspected using the MCD™ Viewer (v1.0.560.6; Fluidigm/Standard BioTools) and exported as 16-bit multichannel TIFF images, with each channel corresponding to a distinct metal isotope. Single-marker images for all 37 markers in paired pre- and post-treatment tissues from patient 0111 are shown in Figures S2 and S3, together with H&E staining and CCR4 immunohistochemistry or CCR4/CD1a dual-color IHC.

### 2.6. IMC Image Processing and Analysis

The resulting TIFF images were imported into Visiopharm Phenoplex (Version 2022.03, Visiopharm A/S, Hørsholm, Denmark) for tissue/cell segmentation, cellular phenotyping, and spatial analysis, following Visiopharm tutorials and protocols [7,8]. Briefly, a machine learning algorithm utilized Collagen I (dermal marker), E-cadherin (epidermal marker), and DNA (nuclear stain) to differentiate between the epidermis, dermis, and non-tissue regions (background/empty space). A pre-trained deep-learning Nuclei Detection AI App was applied to the Ir191/Ir193 DNA intercalation channels for nuclei and cell-body identification. During supervised analysis, all markers were visually inspected, and positivity thresholds were adjusted to minimize batch variation and interference from hot pixels and noise. The lower and upper threshold of each marker in 5 groups are provided in Table S3. Following segmentation and phenotyping in Visiopharm Phenoplex, the resulting data and outputs were exported as standard-format TSV files for downstream analysis. Our immune cell classification strategy first classified cells to six major immune populations (CD4^+^ T cells, CD8a^+^ T cells, CD20^+^ B cells, CD68^+^ macrophages, CD11c^+^ dendritic cells, and CD14^+^ monocytes), and then performed a focused analysis within selected major populations (CD4^+^ T cells, CD68^+^ macrophages, and CD11c^+^ dendritic cells) to resolve phenotypically distinct subsets. This hierarchical approach preserved interpretability at the major-lineage level while enabling finer resolution of within-lineage heterogeneity in biologically relevant compartments.

For unsupervised analysis, IMC OME-TIFF images were normalized using PENGUIN [9], processed in Visiopharm, and exported as single-cell matrices. The resulting data were filtered in R (v4.4) and clustered using Seurat (v5.3.0) following log normalization, principal component analysis (∼10 PCs), and Louvain clustering, with cell types assigned based on marker expression [9,10]. Twenty-nine markers (CD1c, CD3, CD4, CD7, CD8a, CD11b, CD11c, CD14, CD20, CD25, CD27, CD30, CD31, CD45, CD45RA, CD45RO, CD68, CD86, CD163, CD103, CD206, Collagen I, FOXP3, HLA-DR, Ki67, IL-13, PD-1, LAG3, and ICOS), along with cell size, were used for analysis. Batch effect correction was performed using ADTnorm [11].

### 2.7. Statistical Analysis

Data analyses were performed using Microsoft Excel, GraphPad Prism 10.6.1 (GraphPad Software, San Diego, CA, USA), R (4.3 and 4.4), and Python (3.12). Total and compartment-specific cell counts were enumerated for the epidermis, dermis, and intraepidermal niche. Unless otherwise specified, all statistical tests were two-sided. A *p*-value < 0.05 was considered statistically significant, and *p* < 0.01 was considered highly significant. Continuous variables (e.g., percentages of marker-positive cell subsets) were summarized as mean (SD) or median (IQR), and group comparisons were performed using Student’s *t*-test or Welch’s *t*-test, as appropriate, or the Mann–Whitney U test for non-normal data. For cases with two ROIs (0106 and 0312), replicate values were averaged prior to group-level comparisons. Paired continuous outcomes (pre- versus post-treatment) were analyzed using paired *t*-tests or Wilcoxon signed-rank tests. Categorical variables (e.g., MF versus SS, responder versus non-responder) were compared using χ^2^ tests or Fisher’s exact tests, and paired binary outcomes were evaluated using McNemar’s test. For families of multiple comparisons (e.g., marker-wise group differences), *p*-values were adjusted using the Benjamini–Hochberg false discovery rate (BH-FDR) procedure.

## 3. Results

### 3.1. Patient Characteristics and Immunohistochemical Findings in MF/SS Lesional Skin Before and After Mogamulizumab Therapy

**Figure 1:**
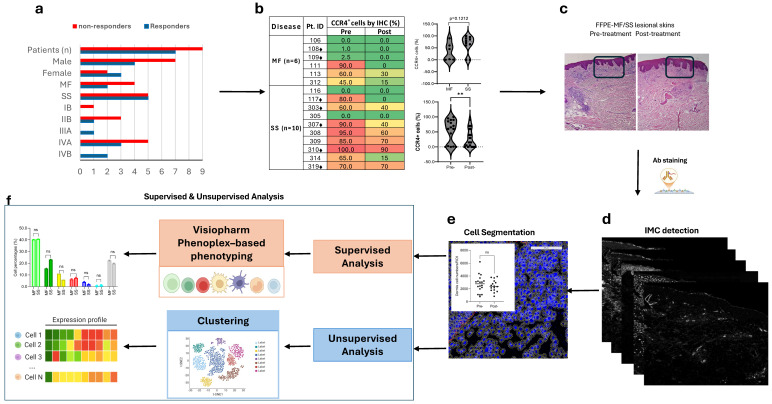
Study cohort, histopathology, and imaging mass cytometry (IMC) workflow. (**a**) Distribution of patient demographics and disease characteristics stratified by responders (blue) and non-responders (red). (**b**) The color-scaled heatmap representation of CCR4^+^ cell percentages in 16 paired lesional skin samples (pre- vs. post-treatment, ^♦^: Responder), stratified by MF and SS; violin plots depict baseline differences between MF and SS and treatment-associated changes. ** *p* < 0.01. (**c**) Representative H&E-stained skin sections with a boxed region of interest (ROI) selected for IMC analysis. (**d**) IMC detection: sequential detection of metal-conjugated antibody signals across tissue sections enables highly multiplexed spatial profiling of lesional skin architecture. (**e**) Cell segmentation: IMC images are computationally processed to delineate individual cells based on nuclear and membrane markers, generating a per-cell dataset of marker expression and spatial features. (**f**) Segmented single-cell datasets are analyzed using both supervised and unsupervised approaches. In the supervised workflow, a Visiopharm Phenoplex-based pipeline assigns cell identities using multiplex marker expression and predefined rules; a representative bar plot summarizes cell population distributions across MF and SS samples. In the unsupervised workflow, dimensionality reduction and clustering (e.g., t-SNE, UMAP) identify cellular populations without prior labeling; an illustrative heatmap depicts marker expression across clusters. Abbreviations: MF, mycosis fungoides; SS, Sézary syndrome; FFPE, formalin-fixed paraffin-embedded; IMC, imaging mass cytometry; H&E, hematoxylin and eosin.

The sixteen patients in this cohort comprised 6 MF patients and 10 SS patients, with a median age of 65.5 years (range 47–86) and a female-to-male ratio of 11:5 (2.2:1) (Table S1, Figure 1a). There were 7 skin responders (7/16. 43.8%) and 9 non-responders. No significant differences in baseline demographics were observed between skin responders and non-responders.

In our previous study, we analyzed CCR4, FOXP3, and CD7 expression in MF/SS lesional skin using immunohistochemistry (IHC) before and after treatment [4]. There were no significant differences in FOXP3 or CD7 expression between pre- and post-treatment lesions, nor between responders and non-responders at baseline. CCR4^+^ cells were higher in SS lesions than in MF (Figure 1b). Following one cycle of mogamulizumab treatment, CCR4^+^ T cells were markedly reduced (Figure 1b), and decreases were observed in 15 of 16 patients, irrespective of clinical response status. Newly performed dual-color IHC evaluating CCR4^+^ cells in conjunction with CD1a^+^ Langerhans cells corroborated these findings (Figure S4). Collectively, these data support on-target depletion of CCR4^+^ cells in lesional skin by mogamulizumab.

CCR4 expression was associated with clinical response. All seven responders had detectable CCR4^+^ T cells at baseline, regardless of expression levels. In contrast, three non-responders lacked detectable baseline CCR4^+^ T cells, while the remaining six exhibited measurable CCR4^+^ T cells in pretreatment lesional skin and showed variable reductions following therapy. These findings suggest that resistance is not determined by CCR4 expression alone but likely reflects additional tumor microenvironment (TME) factors that constrain therapeutic efficacy in the skin. Thus, although mogamulizumab effectively depletes CCR4^+^ cells in lesional tissue, baseline CCR4 expression by itself is insufficient to predict clinical response, highlighting the complexity of the TME in MF/SS.

### 3.2. Immune Cell Profiling in MF/SS Lesional Skin Before and After Mogamulizumab Therapy by Image Mass Cytometry

To further characterize the TME of MF/SS lesions, we utilized imaging mass cytometry (IMC) with a panel of 37 metal-tagged antibodies directed against immune cell subsets and structural components (Table S2). Serial sections stained with H&E confirmed MF/SS histopathology and guided the selection of regions of interest (ROIs) (Figure 1c), focusing on areas with dense cellular infiltration. One ROI was selected per tissue section, except for Patient 0312, from whom two ROIs were obtained from the same pre-treatment section as indicated in Figure S1, and Patient 0106, for whom two ROIs originated from two distinct pre-treatment samples. In total, 34 ROIs were analyzed, including 18 from pre-treatment tissues and 16 from post-treatment tissues.

The 37-marker panel encompassed markers for diverse immune cell types and functional states, as well as markers for epidermal, stromal, and vascular compartments (Table S2). Thirty antibodies were successfully validated and demonstrated consistent performance across samples (Figure 1d).

A total of 68,974 cells was identified across 18 ROIs of pre-treatment tissues, including 49,723 dermal cells, 18,876 epidermal cells, and 384 intraepidermal niche cells, based on nuclear signals from the Iridium-193-labeled DNA intercalator. In comparison, 58,852 cells were identified across 16 ROIs of post-treatment tissues, comprising 39,392 dermal, 19,096 epidermal, and 364 niche cells. Although the total and dermal cell counts were lower in post-treatment tissues, the differences were not statistically significant (Figure 1e). Dermal cells accounted for 71% (70.9% ± 16.2%) of total cells in pre-treatment tissues and 66% (66.3% ± 14.5%) in post-treatment tissues.

**Figure 2:**
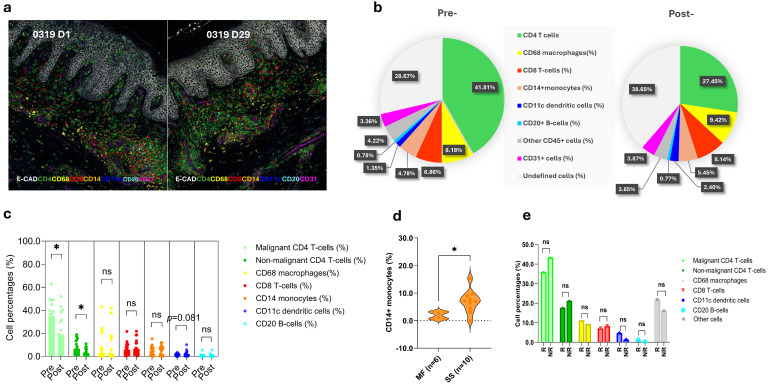
Major immune populations by supervised phenoplex analysis in MF/SS lesional skin before and after mogamulizumab treatment. (**a**) Eight-color IMC overlay images of lesional skin from patient 0319 at Day 1 and Day 29, showing epithelial, endothelial, and immune cell markers (E-cadherin, CD31, CD4, CD8, CD68, CD14, CD11c, CD20). (**b**) Pie charts depicting the relative proportions of major cell populations in pre- and post-treatment MF/SS lesional skin, including CD4^+^ T cells, CD8a^+^ T cells, CD68^+^ macrophages, CD11c^+^ dendritic cells, CD14^+^ monocytes, and CD20^+^ B cells. (**c**) Changes in the major cell populations across all pre- and post-treatment lesional skin (n = 16), distinguishing malignant CD4^+^ T cells from non-malignant CD4^+^ T cells. (**d**) Differences in CD14^+^ monocytes between MF (n = 6) and SS (n = 10) lesional skin before treatment. (**e**) Comparison of the major cell populations between responders (Rs) and non-responders (NRs) before treatment. Statistical significance is indicated as *p* < 0.05 (*); ns = not significant. Abbreviations: IMC, Imaging Mass Cytometry; MF, Mycosis Fungoides; SS, Sézary Syndrome.

In addition to fibroblasts and endothelial cells, we classified immune cells into six major populations using Phenoplex-based supervised annotation: CD4^+^ T cells, CD8a^+^ T cells, CD20^+^ B cells, CD68^+^ macrophages, CD11c^+^ dendritic cells, and CD14^+^ monocytes (Figure 1f). We then performed within-lineage analyses of CD4^+^ T cells, CD68^+^ macrophages, and CD11c^+^ dendritic cells to delineate phenotypically distinct subsets. Eight-color IMC overlay images of paired pre- and post-treatment lesional tissue ROIs from 16 patients were presented in Figure S5. Representative paired images from patient 0319 are presented in Figure 2a. Cell frequencies among dermal cells were quantified at both time points. Mean pre- versus post-treatment frequencies were shown in Figure 2b (CD4^+^ T cells, 41.81% vs. 27.45%; CD68^+^ macrophages, 8.18% vs. 9.42%; CD8a^+^ T cells, 6.86% vs. 8.14%; CD14^+^ monocytes, 4.78% vs. 5.45%; CD11c^+^ dendritic cells, 1.35% vs. 2.40%; and CD20^+^ B cells, 0.78% vs. 0.77%).

Within the CD4^+^ T-cell compartment, malignant T cells were defined by reduced or absent CD3 and/or CD4 expression, loss of CD7, and increased nuclear area (55.46 ± 6.14 μm^2^). Before treatment, these cells comprised the predominant fraction (33.45 ± 14.01%) relative to non-malignant CD4^+^ T cells (8.48 ± 6.18%; unpaired *t*-test, *p* < 0.001). After treatment, both malignant T cells (22.40 ± 14.56%) and non-malignant CD4^+^ T cells (4.11 ± 3.29%) declined significantly (paired *t*-test, *p* < 0.05; Figure 2c). Among antigen-presenting/myeloid subsets, CD11c^+^ dendritic cells trended upward (pre: 1.35 ± 0.98% vs. post: 2.40 ± 2.56%; paired *t*-test, *p* = 0.081; Figure 2c), with similar non-significant increases observed for CD68^+^ macrophages, CD8^+^ T cells, and CD14^+^ monocytes. CD20^+^ B cell frequency showed little change. A portion of dermal CD45^+^ cells was not assigned to the defined lineages (“other CD45^+^ cells”: pre, 4.22 ± 2.27%; post, 3.85 ± 2.09%). The frequency of CD31^+^ endothelial cells changed minimally (pre, 3.38 ± 2.53%; post, 3.87 ± 2.58%; Figure 2b). Unsupervised clustering mirrored the overall shift observed after treatment, with reduced CD4^+^ T cell subsets and increased myeloid cells (dendritic cells and macrophages) (Figure S6).

In pre-treatment tissues, CD14^+^ monocytes were significantly more frequent in SS lesional skin (7.01 ± 3.92%) than in MF (1.93 ± 1.21%; unpaired *t*-test, *p* = 0.012; Figure 2d). Comparing pre-treatment samples from responders (n = 7) and non-responders (n = 9), responders showed lower frequencies of malignant and non-malignant CD4^+^ T cells and modestly higher frequencies of CD11c^+^ dendritic cells and CD20^+^ B cells. However, none of these differences reached statistical significance (Figure 2e).

### 3.3. Immunophenotypic Characterization of Malignant T Cells in MF/SS Lesional Skin Before and After Mogamulizumab Therapy

We next characterized T cell subsets by incorporating additional immunophenotypic markers to delineate distinct functional states and differentiation subsets. These included CD45RO^+^ memory T cells, CD45RA+ naïve T cells, IL-13^+^ type 2 helper T cells (Th2), CD25^+^ activated T cells, FOXP3^+^ regulatory T cells (Treg), CD27^+^ central memory T cells (TCM), CD103^+^ tissue-resident memory T cells (TRM), Ki67^+^ proliferating T cells, CD30^+^ T cells, and co-stimulatory (ICOS) or co-inhibitory molecule (PD-1 and LAG3) expression.

**Figure 3:**
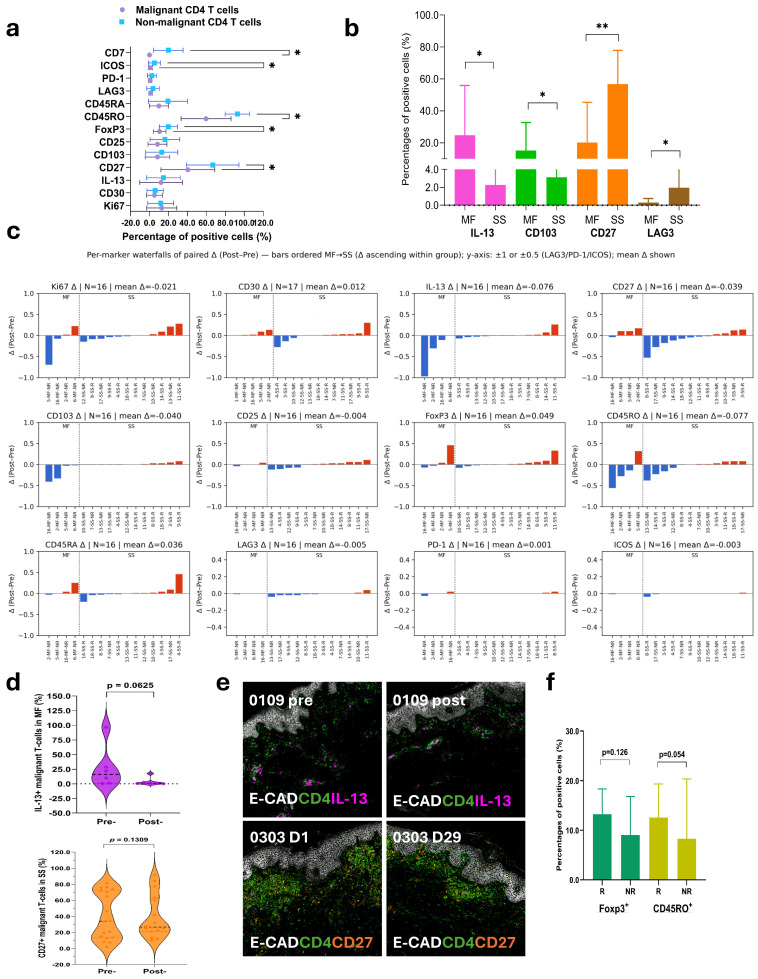
Immunophenotypic features of malignant T cells in MF/SS lesional skin before and after mogamulizumab treatment. (**a**) Functional states and subset profiles of malignant versus non-malignant CD4^+^ T cells across all baseline tissues (n = 16; mean ± SD; *p* values adjusted using the Benjamini–Hochberg procedure; * *q* < 0.05). (**b**) Baseline frequencies of IL-13^+^, CD103^+^, CD27^+^, and LAG3^+^ malignant T-cell subsets between MF and SS lesional skin (mean ± SD; two-sided exact Mann–Whitney U test; * *p* < 0.05, ** *p* < 0.01). (**c**) Waterfall plots showing per-marker change from pre- to post-treatment (Δ = post minus pre); decreases in blue and increases in red; markers ordered within MF and SS by magnitude of change. (**d**) Violin plots of IL-13^+^ and CD27^+^ malignant T-cell subsets in MF and SS pre- and post-treatment tissues. (**e**) Representative IMC images of dermal CD4 (green) co-expressing IL-13 (magenta) or CD27 (orange) in MF (patient 0109, pre- and post-treatment) and SS (patient 0303, Day 1 and Day 29) lesional skin. (**f**) Pre-treatment comparison of FOXP3^+^ and CD45RO^+^ malignant T-cell subsets between responders (Rs) and non-responders (NRs) (mean ± SD; two-sided exact Mann–Whitney U test; exact *p* values annotated). Abbreviations: MF, Mycosis Fungoides; SS, Sézary Syndrome; IMC, Imaging Mass Cytometry.

Comparative analyses of malignant and non-malignant T cell populations were first performed in pretreatment lesions. Approximately 20% of non-malignant T cells expressed CD7 (19.99 ± 15.58%), whereas malignant T cells exhibited markedly reduced or absent CD7 expression (Figure 3a), consistent with prior reports of CD7 loss in CTCL [12]. Among malignant T cells, the fractions of CD27^+^, FOXP3^+^, CD45RO^+^, and ICOS^+^ cells were significantly lower than in their non-malignant counterparts (Figure 3a, Benjamini–Hochberg FDR *q* < 0.05), indicating profound dysregulation of activation, functional, and regulatory pathways within the malignant population. In contrast, the proportions of IL-13^+^, PD-1^+^, LAG3^+^, CD30^+^, and Ki67^+^ cells showed minimal or no differences between malignant and non-malignant T cells (Figure 3a), likely reflecting the immunosuppressive and Th2-skewed microenvironment characteristic of CTCL. Collectively, these findings suggest that malignant T cells share some phenotypic features with non-malignant T cells but exhibit distinct immunophenotypic alterations that may serve as diagnostic indicators.

When comparing malignant T cell phenotypes in pre-treatment lesional skin between MF (n = 8 ROIs) and SS (n = 10 ROIs), IL-13^+^ and CD103^+^ baseline fractions were higher in MF lesions (IL-13^+^: MF: 24.72 ± 31.23%, SS: 2.27 ± 2.69%; *p* = 0.0477; CD103^+^: MF: 15.10 ± 16.09%, SS: 3.10 ± 4.38%; *p* = 0.0159, Figure 3b). Conversely, SS lesions had significantly higher levels of CD27^+^ fractions (SS: 56.77 ± 21.21%, MF: 20.11 ± 25.29%; *p* = 0.0113) and greater LAG3^+^ fractions (SS: 1.95 ± 2.43%, MF: 0.31 ± 0.42%; *p* = 0.0335). These findings underscore distinct immunophenotypic profiles between MF and SS, suggesting subtype-specific mechanisms of immune regulation and potential therapeutic targets.

Paired analyses of pre- and post-treatment tissues revealed a downward trend in IL-13^+^ malignant T cells (Pre: 12.25 ± 22.52% versus Post: 4.17 ± 7.41%; *p* = 0.132), with a more pronounced reduction in MF lesions (Pre: 24.72 ± 31.23% versus Post: 3.76 ± 6.86%; *p* = 0.0625, Figure 3c–e). CD27^+^ malignant T cells also trended downward in SS lesions (Pre: 56.77 ± 21.21% versus Post: 46.07 ± 23.21%; *p* = 0.1309, Figure 3c–e), and LAG3^+^ malignant T cells showed a non-significant decrease (Pre: 1.22 ± 1.74% versus 0.95 ± 1.57%, *p* = 0.115). Other marker changes were varied and not significant (Figure 3c). Comparison of pre-treatment tissues between responders (n = 7) and non-responders (n = 9) revealed slightly lower Ki67^+^, IL-13^+^, CD103^+^, and LAG3^+^ cells and slightly higher CD45RO^+^, and FOXP3^+^ cells (Figure 3f) in responders, though none reached statistical significance. These trends suggest potential immunophenotypic shifts after therapy, warranting further investigation in a larger cohort.

### 3.4. Macrophage Phenotypes and States in MF/SS Lesional Skin Before and After Mogamulizumab Therapy

**Figure 4:**
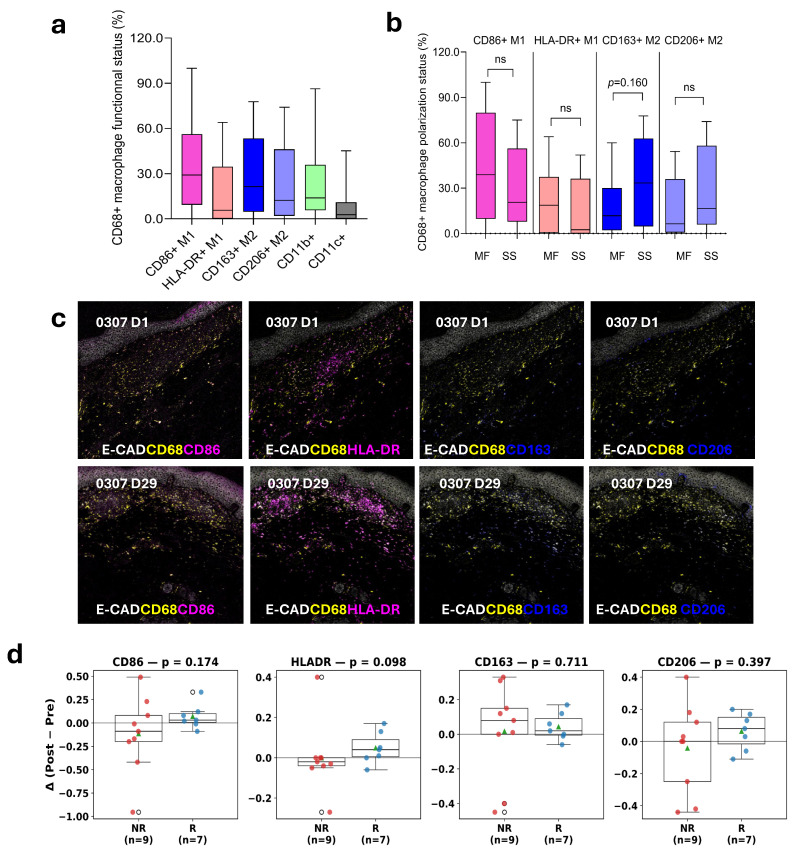
Macrophage phenotypes and states in MF/SS lesional skin before and after mogamulizumab therapy. (**a**) Bar graphs showing the percentage of CD68^+^ macrophages expressing functional markers associated with M1-like (CD86, HLA-DR) and M2-like (CD163, CD206) phenotypes, as well as CD11b and CD11c, across all mycosis fungoides (MF) and Sézary syndrome (SS) lesions. (**b**) Comparison of macrophage polarization status between MF and SS lesions, indicating no significant differences for most markers (ns), with a trend toward higher CD163^+^ M2-like macrophages in SS (*p* = 0.160). (**c**) IMC images of SS skin tissues from patient 0307 at Day 1 (pre-treatment) and Day 29 (post-treatment). The panels display E-cadherin (white), CD68 (yellow) co-localized with CD86 or HLA-DR (magenta), and CD68 (yellow) co-localized with CD163 or CD206 (blue). (**d**) Box plots depicting changes (Δ, post-treatment minus pre-treatment) in CD86, HLA DR, CD163, and CD206 between responders (Rs) and non-responders (NRs). Mann–Whitney *p* values (R vs. NR) are indicated.

CD68^+^ macrophages accounted for a mean of 8.18% of dermal cells at baseline in this cohort (8.18 ± 11.79%; Figure 2b). Within the CD68^+^ macrophage population, expression of polarization-associated markers varied: CD86 (M1-like) was detected in 35.93 ± 31.98% of cells, HLA-DR (M1-like) in 17.49 ± 21.33%, CD163 (M2-like) in 27.95 ± 26.44%, and CD206 (M2-like) in 22.22 ± 24.68%, with expected overlap among these subsets (Figure 4a). CD68^+^ macrophages also expressed CD11b and CD11c.

Pre-treatment MF lesional skin showed higher proportions of CD86^+^ and HLA-DR^+^ M1-like macrophages (CD86^+^, 41.97 ± 37.07%; HLA-DR^+^, 21.62 ± 22.56%; n = 6) compared with SS lesional skin (CD86^+^, 31.09 ± 28.37%; HLA-DR^+^, 14.19 ± 10.88%; n = 10; Figure 4b). In contrast, SS lesions were enriched for CD163^+^ and CD206^+^ M2-like macrophages (CD163^+^, 35.60 ± 29.36%; CD206^+^, 27.34 ± 27.20%) relative to MF lesions (CD163^+^, 18.39 ± 20.03%; CD206^+^, 15.82 ± 21.06%). Consistent with these findings, the mean ratio of CD86^+^ M1-like to CD163^+^ M2-like macrophages was higher in MF than in SS (2.28 versus 0.87). Across pre-treatment samples, M1- and M2-like macrophage distributions varied between responders and non-responders, and notably, the highest CD86^+^-to-CD163^+^ ratios were observed in two responders: one with MF (patient 0108, MF: CD86^+^ 46.81% versus CD163^+^ 10.64%, ratio = 4.4) and one with SS (patient 0307, SS: CD86^+^ 75.0% versus CD163^+^ 6.25%, ratio = 12; Figure 4c).

Following treatment, the overall frequency of CD68^+^ macrophages showed a modest, non-significant increase (Figure 2b,c). Phenotypic changes within the macrophage compartment were heterogeneous across samples. When stratified by clinical skin response, responders exhibited numerically greater increases in CD86^+^ and HLA-DR^+^ M1-like macrophages (Figure 4d), whereas non-responders tended to show decreases in these subsets. CD163^+^ M2-like macrophages changed in both directions in both groups, with particularly wide variability among non-responders. CD206^+^ macrophage proportions trended upward in responders but remained highly variable in non-responders. Consistent with these patterns, the mean ratio of CD86^+^ M1-like to CD163^+^ M2-like macrophages remained higher in responders both before and after treatment (pre, 2.85; post, 2.85) than in non-responders (pre, 0.92; post, 0.90). However, none of the between-group comparisons reached statistical significance in this cohort (CD86^+^, *p* = 0.174; HLA-DR^+^, *p* = 0.098; CD163^+^, *p* = 0.711; CD206^+^, *p* = 0.397; exact two-sided Mann–Whitney U test; Figure 4d), consistent with the limited sample size. Our unsupervised clustering recapitulated higher increases in myeloid M1-like macrophages than myeloid M2-like macrophages after treatment (Figure S6). Collectively, these findings suggest that differences in macrophage polarization may be associated with skin response to mogamulizumab at baseline and/or during treatment, although this trend did not reach statistical significance in this cohort.

### 3.5. Myeloid Dendritic Cell Phenotypes and Subsets in MF/SS Lesional Skin Before and After Mogamulizumab Therapy

CD11c^+^ myeloid dendritic cells (mDCs) comprised multiple subsets, including CD1c^+^ conventional DC2s (cDC2s), CD14^+^ DC3-like cells (often considered a monocyte-associated DC subset), and CD141^+^ conventional DC1s (cDC1s) [13,14,15]. In healthy skin, CD1c^+^ cDC2s predominate among CD11c^+^ mDCs and localize primarily to the dermis, consistent with roles in antigen presentation and immune surveillance, whereas CD14^+^ DC3-like cells are less abundant at steady state and are more closely linked to inflammatory programs and tissue remodeling. Unless otherwise specified, dermal population frequencies are summarized as mean ± SD, whereas marker positivity and subset fractions within the CD11c^+^ compartment are reported as median (95% CI).

**Figure 5:**
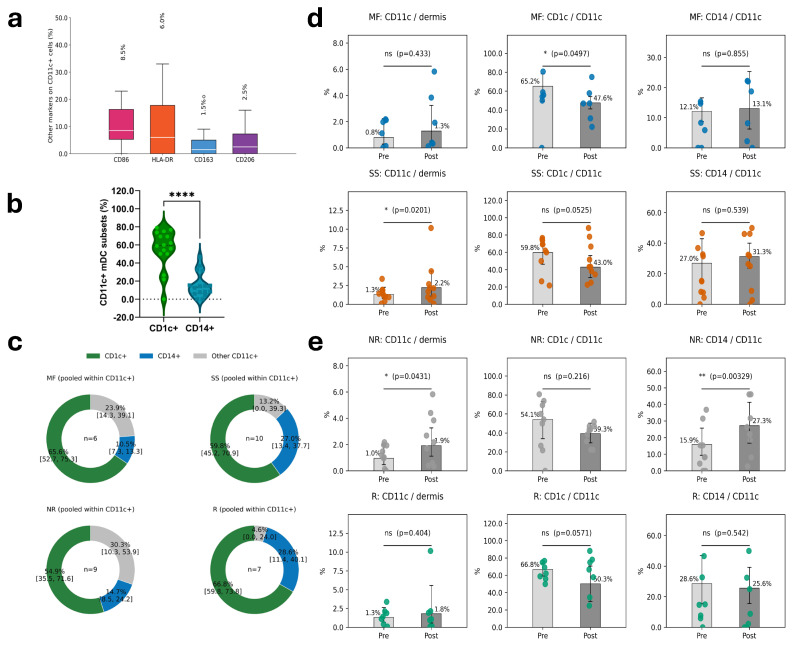
Phenotypes and states of dermal CD11c^+^ myeloid dendritic cells (mDCs) in MF/SS lesional skin at baseline and on-treatment dynamics by disease subtype and clinical response. (**a**) Baseline box plots for co-expression of CD86, HLA-DR, CD163, and CD206 on CD11c^+^ mDCs (medians, 95% CI). (**b**) Violin plots depicting baseline composition within CD11c^+^ mDCs: CD1c^+^ predominance over CD14^+^ (Wilcoxon, *p* = 6.6 × 10^−4^). (**c**) Baseline donut charts (pooled within group) showing CD1c^+^, CD14^+^, and other CD11c^+^ by disease (MF, SS) and response (R, NR). (**d**) Disease-stratified pre/post changes: CD11c^+^/dermis, CD1c^+^/CD11c^+^, CD14^+^/CD11c^+^. Overall CD11c^+^ increased post-treatment, more evident in SS. (**e**) Response-stratified changes: non-responders show CD14^+^ accumulation within CD11c^+^; responders show no CD14^+^ increase. Unless noted, values are percentages; subset proportions are within the CD11c^+^ gate. Points indicate subjects; exact *p*-values are annotated. Abbreviations: MF, mycosis fungoides; SS, Sézary syndrome; R, responder; NR, non-responder. * *p* < 0.05, ** *p* < 0.01, **** *p* < 0.0001.

At baseline, CD11c^+^ myeloid dendritic cells (mDCs) comprised 1.35 ± 0.98% of dermal cells (maximum observed, 3.39%; Figure 2b). Within the CD11c^+^ compartment, the fractions of cells positive for CD86 (median 8.5% [95% CI, 5.5–16.0%]), HLA-DR (median 6.0% [95% CI, 0.0–17.0%]), CD206 (median 2.5% [95% CI, 0.0–7.0%]), and CD163 (median 1.5% [95% CI, 0.0–5.5%]) were generally low (Figure 5a). Subset analysis indicated that the baseline CD11c^+^ mDC compartment was cDC2-dominant (CD1c^+^ cDC2; median 59.4%), whereas CD14^+^ DC3-like cells were less frequent (median 13.4%; Wilcoxon signed-rank test, *p* < 0.001; Figure 5b).

In pretreatment lesions, total CD11c^+^ mDC frequencies were similar between SS and MF skin (SS: 1.38 ± 0.87% vs. MF: 1.33 ± 1.10%). Within the CD11c^+^ mDC compartment, SS showed a trend toward a higher fraction of CD14^+^ DC3-like cells, while CD1c^+^ cDC2 proportions were comparable between disease groups (Figure 5c). When stratified by clinical response, responders exhibited modestly higher baseline CD11c^+^ mDC frequencies than non-responders (1.49 ± 1.11% vs. 1.21 ± 0.78%), although variability was substantial and distributions overlapped. Responders also tended to show higher baseline fractions of both CD1c^+^ cDC2 and CD14^+^ DC3-like cells (Figure 5c).

Following treatment, CD11c^+^ mDC frequencies trended upward (1.35 ± 0.98% pretreatment vs. 2.45 ± 2.56% posttreatment; paired *t*-test, *p* = 0.081; Figure 2c), with a more pronounced increase in SS (Figure 5d). Notably, non-responders demonstrated both an increase in total dermal CD11c^+^ mDCs and a relative accumulation of CD14^+^ DC3-like cells within the CD11c^+^ compartment, whereas responders did not show a corresponding rise in the CD14^+^ DC3-like cell fraction (Figure 5e). In contrast, CD1c^+^ cDC2 fractions were unchanged or tended to decline. An orthogonal unsupervised analysis reproduced the major CD11c^+^ subsets and confirmed the posttreatment increase in DC3-like cells (Figure S6).

Collectively, these data indicate that MF/SS lesional skin is characterized at baseline by a CD1c^+^ cDC2–predominant CD11c^+^ mDC compartment. Treatment was associated with a trend toward increased CD11c^+^ mDC abundance, consistent with increased myeloid influx and/or retention within lesions. Notably, the post-treatment behavior of CD14^+^ DC3-like cells differed by clinical response, with relative enrichment in non-responders. These observations suggest that CD14^+^ DC3-like cell dynamics within the CD11c^+^ mDC compartment may be associated with non-response and merit validation in larger, adequately powered cohorts.

## 4. Discussion

In summary, we used imaging mass cytometry (IMC) to map the cellular composition, immunophenotypic states, and spatial architecture of MF/SS lesional skin before and after mogamulizumab treatment. This single-cell spatial atlas suggests that Th2-skewed T cell programs and myeloid polarization are associated with clinical skin response, while also delineating immune features shared between MF and SS as well as subtype-specific patterns. Our findings indicate that therapeutic efficacy is shaped not only by CCR4 expression on malignant and regulatory T cells, but also by the broader cutaneous tumor microenvironment. Notably, Th2-oriented malignant states, the balance of M1/M2-like macrophage programs, and dendritic-cell composition tracked closely with disease subtype and treatment response, highlighting spatially resolved candidate biomarkers for patient stratification and providing a rationale for combination strategies to improve cutaneous efficacy.

Beygi et al. reported that loss of CCR4 expression and CCR4 genomic alterations can underlie resistance to mogamulizumab in a subset of MF/SS patients [16]. They also documented resistance in individuals without identifiable CCR4 alterations, suggesting that additional mechanisms contribute to treatment failure. Consistent with these observations, three patients in our cohort lacked CCR4 expression at baseline and did not respond to mogamulizumab. Six remaining resistant cases had detectable CCR4 expression. Our findings suggest that microenvironmental features within lesional skin may modulate cutaneous efficacy and contribute to resistance, even when CCR4^+^ cells are present.

In our study, disease subtype-specific differences were apparent: MF lesions were enriched with IL-13^+^ malignant T cells and CD103^+^ tissue-resident memory (TRM) cells, whereas SS lesions contained a higher proportion of CD27^+^ malignant T cells with elevated LAG3 expression. Despite these distinctions, both MF and SS demonstrate a global Th2-dominant milieu that likely blunts anti-tumor immunity. Although malignant CD4^+^ T cells exhibit broad dysregulation compared with non-malignant CD4^+^ cells, a pervasive Th2 bias and increased expression of co-inhibitory receptors (PD-1, LAG3) are present across both malignant and non-malignant T cells, reflecting the immunosuppressive tumor microenvironment in MF/SS. These results are consistent with Li et al., who reported that malignant CTCL cells co-opt Th2-associated gene programs and remodel the tumor microenvironment to support disease persistence [17].

**Figure 6:**
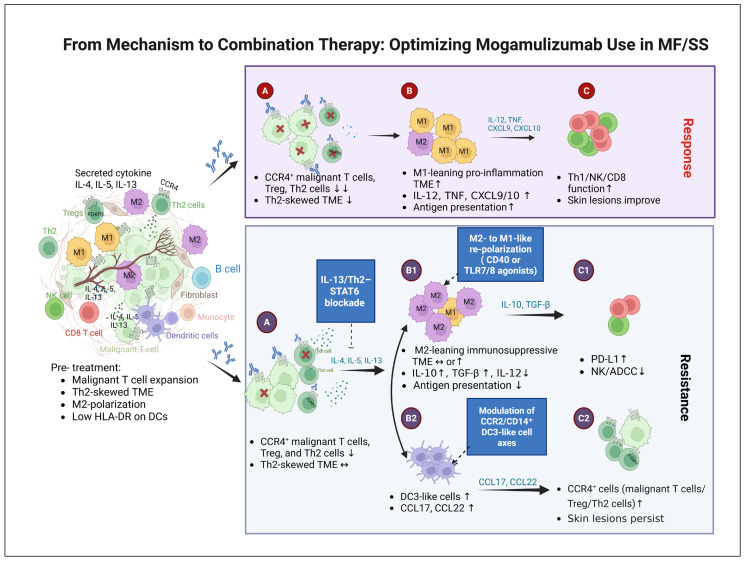
Proposed mechanistic model and combination strategies for mogamulizumab therapy. Schematic overview of the tumor microenvironment prior to treatment, characterized by a Th2-skewed immune milieu, predominant M2 macrophage polarization, and reduced dendritic cell (DC) HLA-DR expression. Clinical response to mogamulizumab is associated with depletion of CCR4^+^ malignant cells and Th2-biased or immunosuppressive cell populations, macrophage repolarization toward an M1-leaning phenotype, enhanced antigen presentation, and increased activation of Th1, NK, and CD8^+^ effector cells. Proposed mechanisms of resistance include incomplete depletion of Th2-biased or immunosuppressive cells, persistent M2 macrophage dominance, DC modulation with impaired antigen presentation, and PD-L1 upregulation, collectively promoting immune evasion. Solid arrows indicate experimentally supported interactions, whereas dashed arrows denote hypothesized pathways and potential targets for rational combination therapies. Symbols: ↑ indicates increased abundance, expression, or activity; ↓ indicates decreased abundance, expression, or activity; ↔ indicates no substantial change. This presentation was created in BioRender (https://BioRender.com).

Both M1- and M2-like macrophages are present in normal skin under homeostatic conditions, with a predominance of M2-like macrophages that supports tissue maintenance and limits unnecessary inflammation [18,19,20]. In contrast, macrophages within the tumor microenvironment (TME) are generally skewed toward an M2-like, immunosuppressive phenotype, whereas M1-like macrophages are relatively infrequent and are typically enriched only in highly inflamed or treatment-responsive tumors. In our study, we identified frequent M1-like macrophages (CD86^+^, HLA-DR^+^) within MF/SS lesions, with MF demonstrating greater M1 enrichment compared with SS lesions, which were more strongly polarized toward an M2-like phenotype (CD163^+^, CD206^+^). These findings are consistent with a chronic inflammatory microenvironment in MF/SS tissues. Importantly, macrophage polarization was associated with clinical outcome: responders exhibited higher baseline M1/M2 ratios and demonstrated post-treatment increases in M1-like macrophages, whereas non-responders showed attenuation of M1-associated features. Supporting these observations, prior studies by Wu et al. demonstrated that M2-like macrophages contribute substantially to CTCL pathogenesis and that depletion of M2-like macrophages in murine models delays tumor development [21,22]. Moreover, Masson et al. recently reported post-mogamulizumab increased in cutaneous macrophages and macrophage-derived chemokines CXCL9 and CXCL11, which were associated with treatment-related skin rash and correlated with prolonged remission [23]. Collectively, these findings support a model in which a pre-existing or therapy-induced inflammatory myeloid microenvironment augments the clinical efficacy of mogamulizumab (Figure 6).

In our cohort, CD11c^+^ myeloid dendritic cells (mDCs) exhibited reduced HLA-DR expression in MF/SS lesions compared with normal skin where dermal CD11c^+^ cells uniformly express HLA-DR [15]. CD1c^+^ conventional type 2 dendritic cells (cDC2s) remained the predominant myeloid DC subset across MF/SS lesions. In parallel, CD14^+^ DC3-like cells were increased, with the most pronounced expansion observed in SS. In fact, a prior work by Nakamizo et al. reported an expansion of CD14^+^ DCs (DC3s) in inflammatory dermatoses, including atopic dermatitis and psoriasis, and implicated this subset in promoting psoriatic inflammation [13]. Following mogamulizumab treatment, total CD11c^+^ dendritic cells increased modestly overall, driven primarily by accumulation of CD14^+^ DC3-like cells in non-responders, whereas responders did not exhibit a comparable increase. This pattern is consistent with findings reported by Li et al., who demonstrated that dendritic cells—predominantly monocyte-derived DC3/CD14^+^ DCs—can support Th2-like malignant cell programs in CTCL [17]. Indeed, CD14^+^ DC3-like cells are highly plastic and exist along a continuum of activation states within inflamed skin and the tumor microenvironment. Rather than representing a fixed or immutable cell type, these cells dynamically adapt their phenotype and function in response to local tissue-derived signals [24,25]. Collectively, these dynamics suggest that enrichment of CD14^+^ DC3-like cells may promote a persistently immunosuppressive microenvironment and attenuate cutaneous responses to therapy. Prospective mechanistic studies are warranted to determine whether CD14^+^ DC3-like cells constitute a critical myeloid node mediating resistance to mogamulizumab.

Based on our findings, we propose a mechanistic model of mogamulizumab therapy (Figure 6). The IL-13-driven Th2-high malignant program signals through IL-4Rα/IL-13Rα1–STAT6 to polarize macrophages toward M2 (e.g., ARG1, MRC1/CD206) and increase IL-10 and TGF-β to suppress IL-12. This shift reduces antigen presentation (↓HLA-DR and costimulatory molecules) and promotes PD-L1 expression [26]. Concurrently, CD14^+^ DC3-like cells conditioned by Th2 cues produce CCL17/CCL22, recruiting CCR4^+^ malignant and regulatory T cells and establishing a feed-forward Th2 loop [13,27]. The resulting milieu impairs NK-cell ADCC (via IL-10, TGF-β, and arginase-mediated arginine depletion) and weakens effective priming. By contrast, lower IL-13 levels with an M1-dominant macrophage profile enhance IL-12, TNF, and CXCL9/10, restore antigen presentation, and support Th1/Tc1 and NK recruitment [28]—conditions that should potentiate mogamulizumab-mediated depletion of CCR4^+^ targets. The association of lower IL-13, higher M1/M2, and lack of DC3 expansion in responders is consistent with this model.

Our study confirms that imaging mass cytometry is a robust method for high-dimensional, spatially resolved profiling of immune and structural elements in FFPE tissues [29]. Our guided phenotyping workflow, using expert-informed thresholds across multiplex markers, facilitated reproducible subset calls across regions and samples. Nevertheless, this study has a few limitations. All post-treatment skin biopsies were collected only after one treatment cycle, and more pronounced or durable changes relevant to efficacy may require longer treatment exposure. Several post-treatment trends did not reach statistical significance and therefore require validation in larger cohorts with extended follow-up. Additional limitations include the suboptimal performance of a few key antibodies, most notably anti-CCR4, which precluded direct, compartment-level correlation of CCR4 expression. After all, this small exploratory study is not powered for definitive conclusions but achieved its intended aims by identifying trends and generating hypotheses for future studies.

Future studies should validate the baseline burden of IL-13^+^ malignant T cells, macrophage polarization status, and the abundance of CD14^+^ DC3-like cells as predictive biomarkers of clinical treatment with mogamulizumab. Incorporating spatial interaction metrics—such as malignant T cell–macrophage and malignant T cell–DC3 adjacency—will refine mechanistic models. It would be highly informative to directly compare malignant T cells and other immune populations in lesional skin and peripheral blood within the same patient cohort, both before and after mogamulizumab treatment. Rational combination strategies should also be tested, including IL-13/Th2–STAT6 pathway blockade [30] and agents that re-polarize macrophages toward M1-like states (e.g., CD40 or TLR7/8 agonists) [31,32] or modulate CCR2/CD14^+^ DC3-like cell axes, with the goal of enhancing cutaneous responses (Figure 6). Finally, standardization of IMC panels and staining workflows across sites will be essential for multicenter reproducibility and clinical translation.

## 5. Conclusions

In MF/SS, mogamulizumab efficacy reflects both target engagement and the lesional immune context: Th2-skewed (IL-13^+^) malignant programs, M2-polarized myeloid states, and expansion of CD14^+^ DC3-like cells are associated with non-response, whereas M1-dominant macrophages and attenuation of Th2 features accompany benefit. These spatial readouts support biomarker-guided patient selection and rational combination strategies.

## Data Availability

Most data are provided within the article and its Supplementary Materials. All data generated in this study are available upon request from the corresponding author.

## Supplementary Materials

The following supporting information can be downloaded at https://www.mdpi.com/article/10.3390/cancers18142348/s1, Table S1: The demographics, malignant T-cells, T_reg_ cells, NK cells, and responses to mogamulizumab in CTCL patients; Table S2: Imaging mass cytometry antibody panel; Table S3: The lower threshold and upper threshold of each marker in 5 groups of tissues; Figure S1: IMC Preview Mode for 5 pairs of tissues; Figure S2: Single-marker IMC images for all 37 markers in pre-treatment tissue (0111); Figure S3: Single-marker IMC images for all 37 markers in post-treatment tissue; Figure S4: Dual-color IHC staining for CCR4 and CD1a in 2 pairs of tissues; Figure S5: Eight-color IMC overlay images in paired pre- and post-treatment lesional skins for 16 patients; Figure S6: Cell clustering and marker-specific expression identified by unsupervised IMC image analysis with differential abundance between pre- and post-treatment samples.

## Abbreviations

The following abbreviations are used in this manuscript:

CTCL	Cutaneous T- cell lymphoma
MF	Mycosis fungoides
SS	Sézary syndrome
TME	Tunor microenvironment
IHC	Immunohistochemistry
IMC	Imaging mass cytometry
ROI	Region of interest
M1-like macrophages	Macrophages that display M1-type markers/functions
M2-like macrophages	Macrophages that display M2-type markers/functions
mDCs	Myeloid dendritic cells
cDC2	conventional dendritic cell type 2
DC3	dendritic cell type 3
