# Supplementary material for: Dissecting Immune Determinants in Lesional Skin of Cutaneous T-Cell Lymphoma During Mogamulizumab Therapy
Source: Cancers (Basel). 2026 Jul 21;18(14):2348. doi: 10.3390/cancers18142348 (PMC13406218; doi:10.3390/cancers18142348)

Table S1 The demographics, malignant T-cells, T_{reg} cells, NK cells, and responses to mogamulizumab in CTCL patients

Primary Diagnosis	Patient ID	Stage	Age* (year)	Gender	Dose/course	Phase	Malignant PB T-cells (%)*	CCR4+ Malignant T-cells (% CCR4)	T_{reg} cells (%)*	CCR4+ T_{reg} cells (% CCR4)	Foxp3 mRNA (fold)	CCR4 mRNA (fold)	NK cells (%)*	CCR4+ cells in skin lesions (%)*	Clinical Response			
															Blood	Skin	Lymph node	Overall
MF (n=6)	106	IIB	73	M	1.0 mg/kg/1	II	0	-	0	-	0.08	0.73	16.4	0	-	SD	-	SD
	108	IIB	66	M	1.0 mg/kg/1	II	0	-	1.6	94.4	0.32	1.13	36.4	1	-	PR	-	PR
	109	IIIA	48	F	1.0 mg/kg/1	II	0	-	2.1	93.8	0.34	2.3	14.9	2.5	-	PR	-	PR
	111	IIB	76	M	1.0 mg/kg/1	II	16.2	3.3	0.7	90.2	0.07	0.64	24.7	90	SD	SD	-	SD
	113	IB	79	M	1.0 mg/kg/1	II	21.2	94.9	2.3	96	0.43	4.16	32.9	60	PR	SD	SD	SD
	312	IIB	53	M	1.0 mg/kg/1	II	13.7	20.8	1.5	58.6	0.29	4.75	-	45	SD	SD	-	SD
**Mean ± SD**			**65**<br>**(48 - 86)**	**5/1**<br>**(M/F)**	**1.0 mg/kg/1**	**II**	**8.5 ± 9.6**	**39.7 ± 48.6**	**1.4 ± 0.9**	**86.6 ± 15.8**	**0.26 ± 0.15**	**2.29 ± 1.79**	**25.1 ± 9.6**	**33.1 ± 37.9**	**1/3**<br>**33.33%**	**2/6**<br>**33.33%**	**0/1**<br>**-**	**2/6**<br>**33.33%**
SS (n=10)	116	IVA	62	F	1.0 mg/kg/1	II	95	95.1	0	-	0.03	12.92	4.8	0	PR	SD	SD	SD
	117	IVB	79	F	1.0 mg/kg/1	II	73.8	97.4	1.5	97.8	0.08	24.68	6	80	PR	SD (PR-C5#)	SD (PD-C5#)	SD (PD-C5#)
	303	IVA	65	F	0.3 mg/kg/1	I	7.7	100	2.5	59.3	-	-	-	60	CR	SD (PR-C3#)	-	SD (PR-C3#)
	305	IVA	47	M	1.0 mg/kg/1	I	58.7	88.3	2.9	97.9	4.06	38.92	7.3	0	SD	SD	SD	SD
	307	IVA	61	M	1.0 mg/kg/1	II	55.2	96.5	0	-	-	-	-	90	CR	SD (PR-C5#)	SD (PR-C5#)	SD (PR-C5#)
	308	IVA	63	M	1.0 mg/kg/1	II	40.9	69.1	1	91.7	0.67	16.99	-	95	SD	SD	PD	SD
	309	IVA	76	M	1.0 mg/kg/1	II	82.3	91.3	0.4	95.7	1.19	63.04	1.3	85	PR	SD	SD	SD
	310	IVA	62	M	1.0 mg/kg/1	II	94.5	99.5	0	-	0.3	134.02	1.3	100	CR	PR	PR	PR
	314	IVA	72	F	1.0 mg/kg/1	II	60.1	96.5	0	-	0.66	7.2	-	65	PR	SD	SD	SD
	319	IVB	86	M	1.0 mg/kg/1	II	86.5	90.5	0.4	97.1	0.47	41.28	12.4	70	PR	SD (PR-C3#)	SD (PR-C3#)	SD (PR-C3#)
**Mean ± SD**			**72**<br>**(47- 79)**	**6/4**<br>**(M/F)**	**1/9**<br>**(0.3/1.0) mg/kg/1**	**2/8**<br>**(I/II)**	**65.5 ± 27.1**	**92.4 ± 9.1**	**0.9 ± 1.1**	**89.9 ± 15.2**	**0.93 ± 1.3**	**42.38 ± 41.23**	**5.5 ± 4.2**	**64.5 ± 36.3**	**8/10**	**1/10 (5/10#)**	**1/10 (3/10#)**	**1/10 (4/10#)**
**P value**							***<0.01***	***0.1 (NS)***	***0.17 (NS)***	***0.37 (NS)***	***0.1 (NS)***	***<0.05***	***<0.01***	***0.07 (NS)***	**80.00%**	**10.00% (50.0%#)**	**10.00% (30.00%#)**	**10.00% (40.4%#)**
**Sum**	**n=16**		**65.5**<br>**(47 - 86)**	**11/5**<br>**(M/F)**	**1/15**<br>**(0.3/1.0) mg/kg/1**	**2/14**<br>**(I/II)**	**44.1 ± 35.8**	**80.2 ± 31.5**	**1.1 ± 1.0**	**88.4 ± 14.8**	**0.64 ± 1.0**	**25.20 ± 36.62**	**14.4 ± 12.2**	***52.7 ± 38.9***	**9/13**<br>**69.23 %**	**3/16 (7/16#)**<br>**18.75% (43.8%#)**	**1/11 (3/11#)**<br>**9.09% （27.27#）**	**3/16 （6/16）**<br>**18.75% (37.5%#)**

*Median (range); # clinical response after Course 3 (C3) or Course 5(C5).

Table S2 Imaging mass cytometry antibody panel

	Target	Clone	Metal	Supplier	Catalog #	Dilution	Incubation
1	CCR4**	Polyclonal/Qdot800*	$^{112}Cd/^{114}Cd$	R&D	NBP1-86584	1:200	4℃, overnight
2	CD1a**	O10	^{115}ln	R&D	NBP2-34697	1:50	4℃, overnight
3	CD69**	8B6	^{141}Pr	R&D	NBP1-51607	1:50	5 hours, RT
4	ICOS	D1K2T	^{142}Nd	CST	39740SF	1:50	5 hours, RT
5	CD123**	8E11C5	^{143}Nd	R&D	NBP2-37354	1:200	4℃, overnight
6	CD14	EPR3653	^{144}Nd	MDACC CORE	3144025D	1:400	4℃, overnight
7	CD7	1G10D8	^{145}Nd	Proteintech	60209-1-IG	1:50	5 hours, RT
8	Collagen I	3G3	^{146}Nd	Abcam	ab88147	1:500	4℃, overnight
9	CD163	EDHu-1	^{147}Sm	MDACC CORE	3147021D	1:800	4℃, overnight
10	CD30	E7E4D	^{148}Nd	CST	18445SF	1:50	5 hours, RT
11	CD11b	EPR1344	^{149}Sm	Standard BioTools	3149028D	1:400	4℃, overnight
12	Ki-67	B56	^{150}Nd	Standard BioTools	91H017150	1:200	4℃, overnight
13	CD103	ITGAE/3904R	^{151}Eu	R&D	NBP3-08855	1:75	5 hours, RT
14	CD45	D9M8I	^{152}Sm	MDACC CORE	3152018D	1:400	4℃, overnight
15	LAG3	BLR027F/BLR028F	^{153}Eu	MDACC CORE	A700-027CF & A700-028CF	1:200	4℃, overnight
16	CD11c	EP1347Y	^{154}Sm	Baylor CORE	NA	1:150	4℃, overnight
17	Foxp3	PCH101	^{155}Gd	MDACC CORE	3155018D	1:50	4℃, overnight
18	CD4	EPR6855	^{156}Gd	MDACC CORE	3156033D	1:150	5 hours, RT
19	E-Cadherin	2.40E+11	^{158}Gd	MDACC CORE	3158029D	1:400	4℃, overnight
20	CD68	KP1	^{159}Tb	MDACC CORE	3159035D	1:400	4℃, overnight
21	CD31	JC/70A	^{160}Gd	R&D	NB600-562	1:100	4℃, overnight
22	CD20	H1	^{161}Dy	MDACC CORE	3161029D	1:200	4℃, overnight
23	CD8a	C8/144B	^{162}Dy	MDACC CORE	3162034D	1:200	4℃, overnight
24	CD56**	EPR2566	^{163}Dy	Baylor CORE	NA	1:600	4℃, overnight
25	CD86	BU63	^{164}Dy	R&D	NBP2-25208	1:150	4℃, overnight
26	PD-1	EPR4877(2)	^{165}Ho	Baylor CORE	NA	1:600	4℃, overnight
27	CD45RA	HI100	^{166}Er	Standard BioTools	3166031D	1:200	4℃, overnight
28	CD1c	OTI2F4	^{167}Er	R&D	NBP2-70345	1:100	4℃, overnight
29	CD206	5C11	^{168}Er	R&D	H00004360-M02	1:250	4℃, overnight
30	IL-13	2B5	^{169}Tm	R&D	H00003596-M07	1:50	4℃, overnight
31	CD3	Polyclonal	^{170}Er	MDACC CORE	3170019D	1:200	5 hours, RT
32	CD27	BLR083G	^{171}Yb	MDACC CORE	A700-083CF	1:800	4℃, overnight
33	CD141**	THBD/1782	^{172}Yb	R&D	NBP2-54496-100ug	1:200	4℃, overnight
34	CD45RO	UCHL1	^{173}Yb	MDACC CORE	3173016D	1:2000	4℃, overnight
35	HLA-DR	LN3	^{174}Yb	MDACC CORE	3174025D	1:3200	4℃, overnight
36	CD25	EPR6452	^{175}Lu	MDACC CORE	3175036D	1:50	5 hours, RT
37	IFNG**	EPR21704	^{176}Yb	Abcam	ab231301	1:75	4℃, overnight

*Qdot800-labeled secondary antibody containing $^{112}Cd/^{114}Cd$; **Failed markers.

Table S3 The lower threshold and upper threshold of each marker in 5 groups of tissues

		Group 2		Group 3a		Group 3b		Group 4		Group 5	
**Channel**	**Marker**	**LT**	**UT**	**LT**	**UT**	**LT**	**UT**	**LT**	**UT**	**LT**	**UT**
Nd(142)	ICOS	0.50	46.88	0.51	3.26	0.60	46.84	0.38	5.06	0.38	5.06
Nd(144)	CD14	3.28	15.63	1.46	8.30	1.33	17.57	0.71	14.38	0.71	14.39
Nd(145)	CD7	0.41	4.97	0.30	1.74	0.28	4.98	0.24	2.99	0.25	2.99
Sm(147)	CD163	0.55	14.54	0.61	6.78	0.58	14.56	0.49	7.53	0.49	7.52
Nd(148)	CD30	1.13	8.37	1.15	5.55	0.30	8.37	0.20	1.38	0.21	1.38
Sm(149)	CD11b	1.20	21.84	1.08	8.67	1.06	21.88	0.52	7.96	0.51	7.96
Nd(150)	Ki67	1.70	77.60	2.24	44.16	1.60	77.52	0.29	34.51	0.25	34.57
Eu(151)	CD103	0.37	15.94	0.27	1.89	0.40	15.97	0.29	6.20	0.29	6.19
Sm(152)	CD45	0.19	11.03	0.26	5.12	0.21	11.05	0.09	5.07	0.11	5.08
Eu(153)	LAG3	1.60	14.97	0.61	11.96	1.72	14.82	0.39	8.21	0.37	8.20
Sm(154)	CD11c	0.64	10.13	0.64	1.84	0.63	11.13	0.44	4.05	0.44	4.04
Gd(155)	FoxP3	0.63	76.43	0.70	6.91	0.57	76.38	0.20	5.08	0.20	5.08
Gd(156)	CD4	1.34	114.73	1.40	12.39	1.33	114.93	0.82	16.14	0.84	16.11
Tb(159)	CD68	2.66	156.16	4.27	78.60	3.44	155.71	6.91	267.49	7.48	267.47
Gd(160)	CD31	1.82	125.27	1.48	16.48	1.79	124.97	0.41	5.27	0.40	5.25
Dy(161)	CD20	1.99	45.26	0.40	1.63	1.95	45.20	0.52	4.79	0.53	4.77
Dy(162)	CD8a	2.29	32.50	0.87	17.20	1.40	32.54	1.31	16.06	1.32	16.02
Dy(164)	CD86	1.42	5.67	1.00	1.56	0.24	6.99	0.22	4.49	0.23	4.49
Ho(165)	PD-1	0.54	1.66	0.36	0.56	0.14	1.66	0.20	0.79	0.20	0.79
Er(166)	CD45RA	1.36	45.35	2.25	44.34	1.42	45.48	0.99	20.79	1.05	20.77
Er(167)	CD1c	0.67	11.13	0.52	4.56	0.69	11.17	0.42	2.96	0.29	7.11
Er(168)	CD206	5.30	190.69	1.14	22.36	4.74	190.23	2.00	259.84	2.69	259.92
Tm(169)	IL-13	0.97	28.11	0.37	7.27	0.63	7.19	0.34	7.84	0.35	7.84
Er(170)	CD3	0.50	9.70	0.36	3.10	0.49	9.67	0.43	8.58	0.44	8.59
Yb(171)	CD27	0.48	10.55	0.21	4.15	0.50	10.58	0.29	6.83	0.31	6.83
Yb(173)	CD45RO	0.46	24.69	0.77	15.18	0.50	24.71	0.82	16.52	0.84	16.50
Yb(174)	HLA-DR	2.91	147.46	10.11	131.05	3.70	147.62	10.19	194.68	9.87	194.64
Lu(175)	CD25	0.54	19.82	0.49	1.40	0.38	19.82	0.15	2.14	0.15	2.14

Group 2: 0109-pre, 0109-post, 0117-pre, 0117-post; **Group 3a:** 0111-pre; **Group 3b:** 0111-post, -106-pre1, 0106-pre2, 0106-post, 0108-pre, 0108-post, 0113-pre, 0113-post, 0116-pre, 0116- post, control T3-6, control T4-7; **Group 4:**03-09-D1, 0309-D29; **Group 5:** 0303D-1,0303-D29, 0305-D1, 0305-D29, 0307-D1, 0307-D29, 0308-D1, 0308-D29, 0310-D1, 0310-D29, 0312-D1, 0312-D1.1, 0312-D1.2, 0312-D29, 0314-D1, 0314-D29, 0319-D1, 0319-D29. LT: Lower Threshold; UT: Upper Threshold.

Figure S1. IMC Preview Mode for 5 pairs of tissues

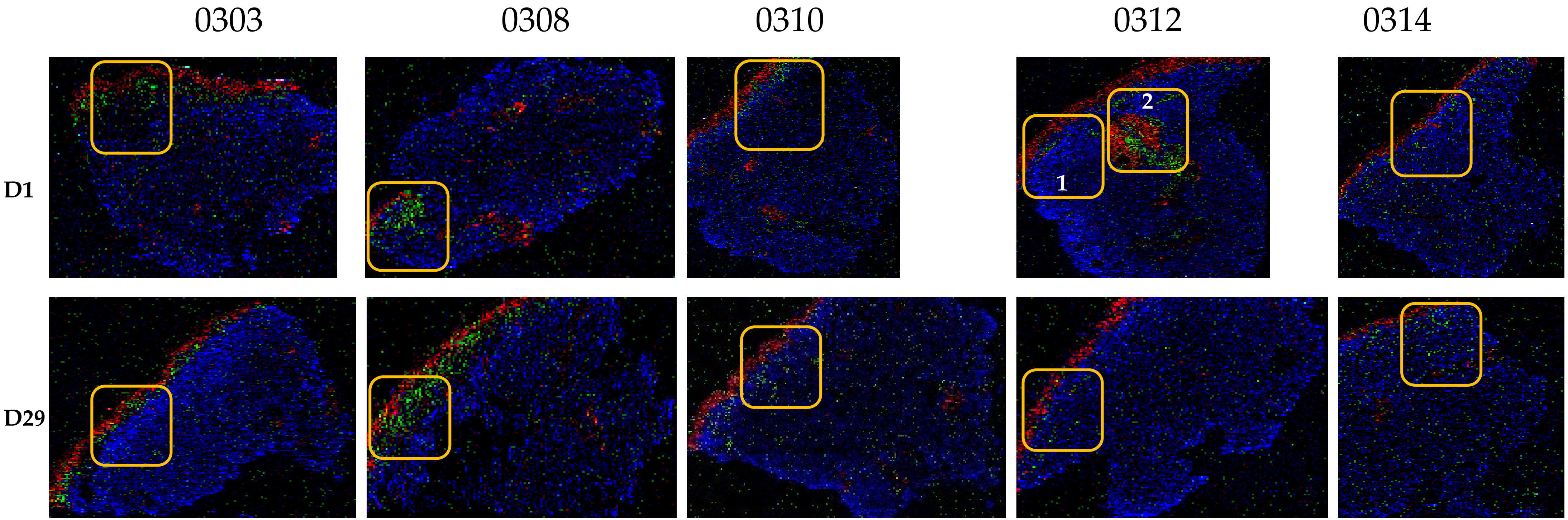

IMC Preview Mode were used for rapid scan of paired tissue sections in 5 patients before full IMC acquisition to identify regions of interest (ROIs, square in orange). Two ROIs for 0312 pre-treatment section (D1: 1 & 2,) were selected as indicated. E-CAD: red; CD45: green; Collagen I: Blue.

Figure S2. Single-marker IMC images for all 37 markers in pre-treatment tissue (0111)

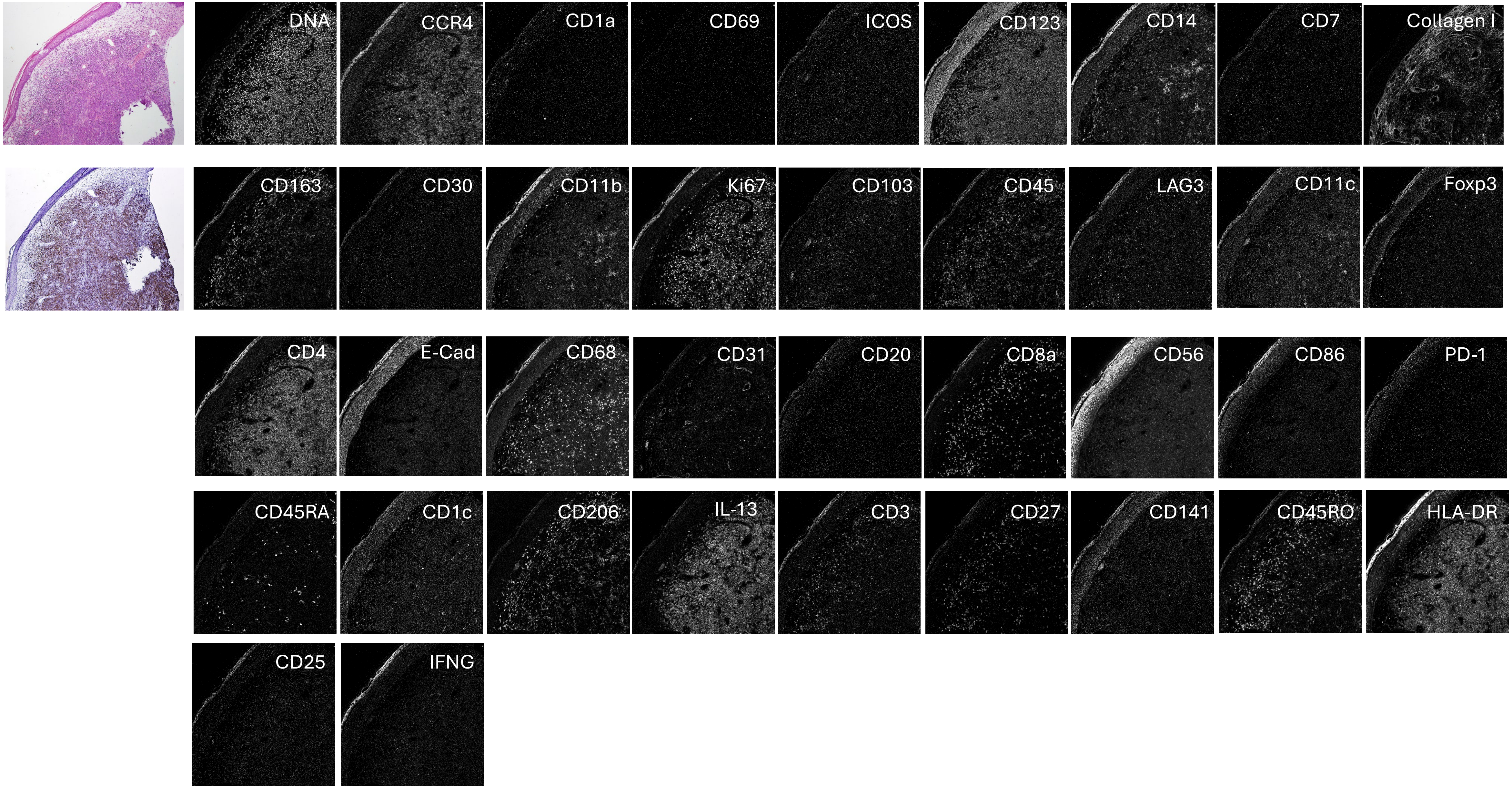

Figure S3. Single-marker IMC images for all 37 markers in post-treatment tissue (0111)

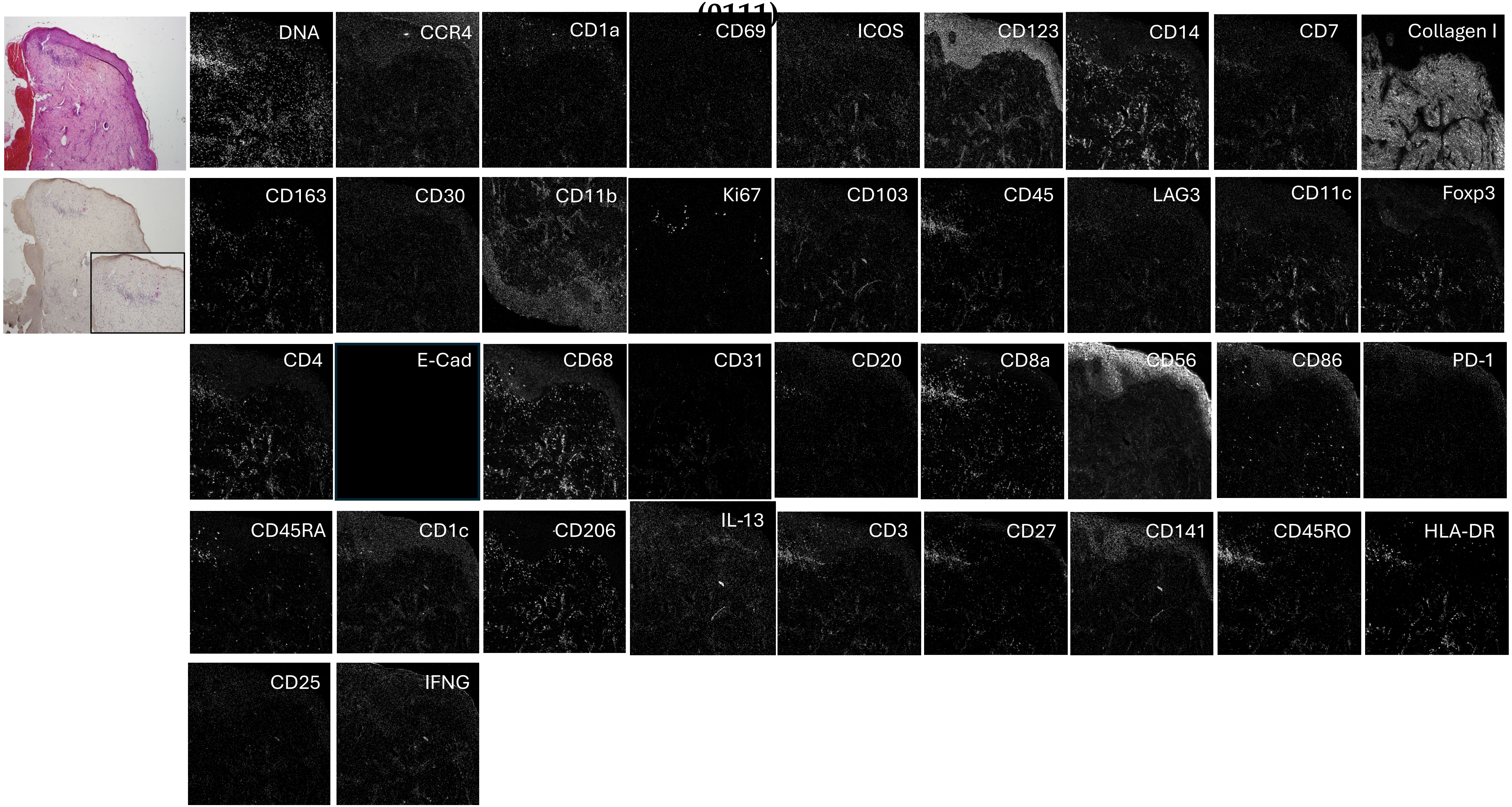

Figure S4. Dual-color IHC staining for CCR4 and CD1a in 2 pairs of tissues

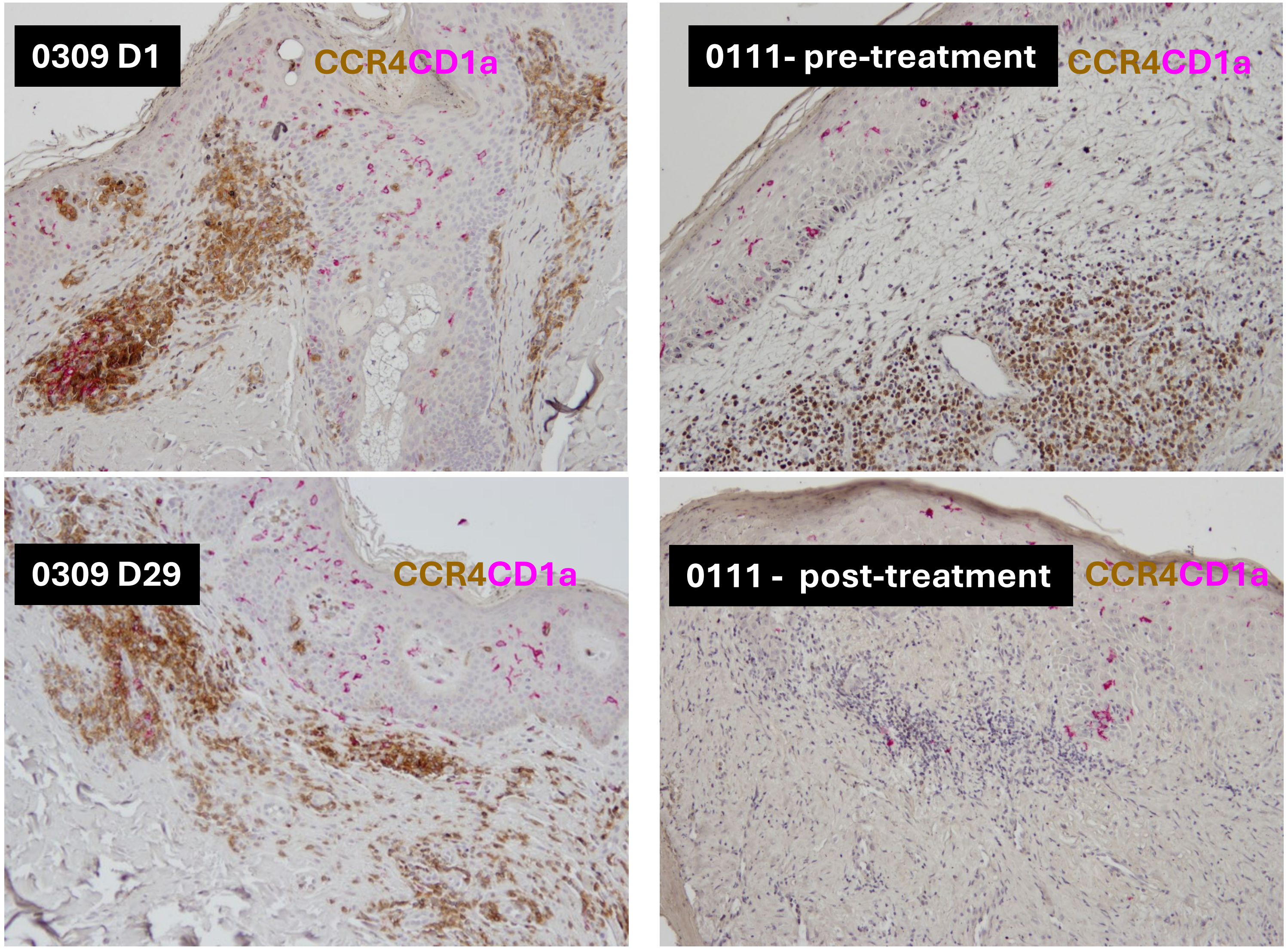

Figure S5. Eight-color IMC overlay images in paired pre- and post-treatment lesional skins for 16 patients

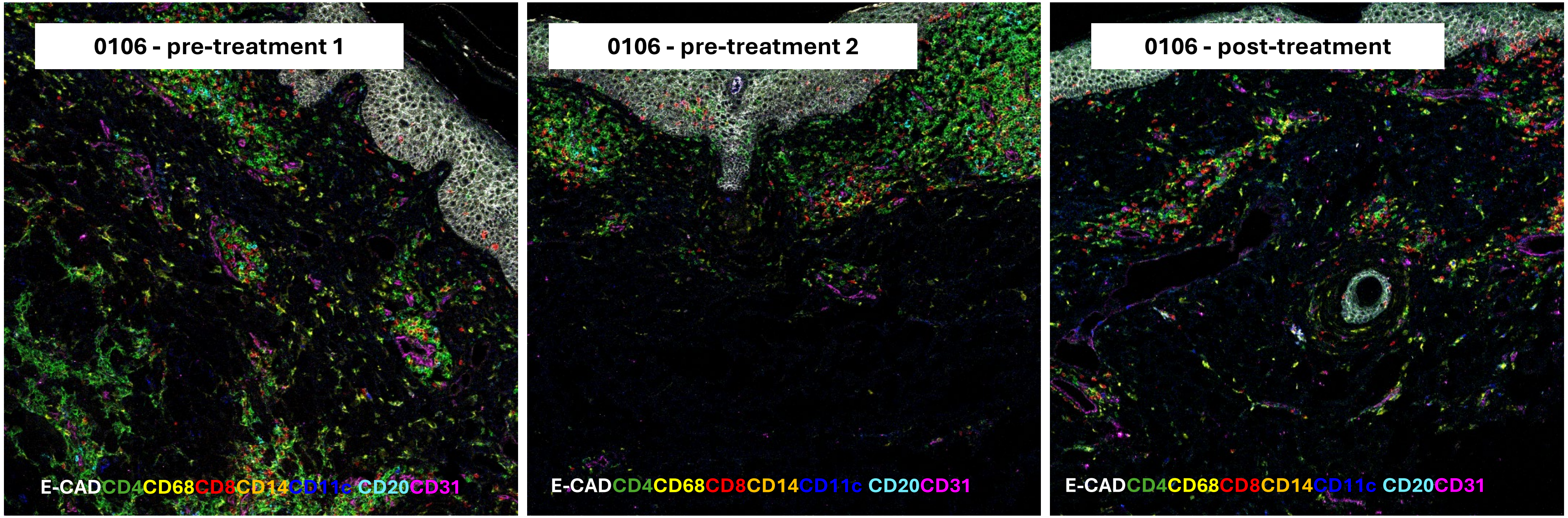

Figure S5. (continued)

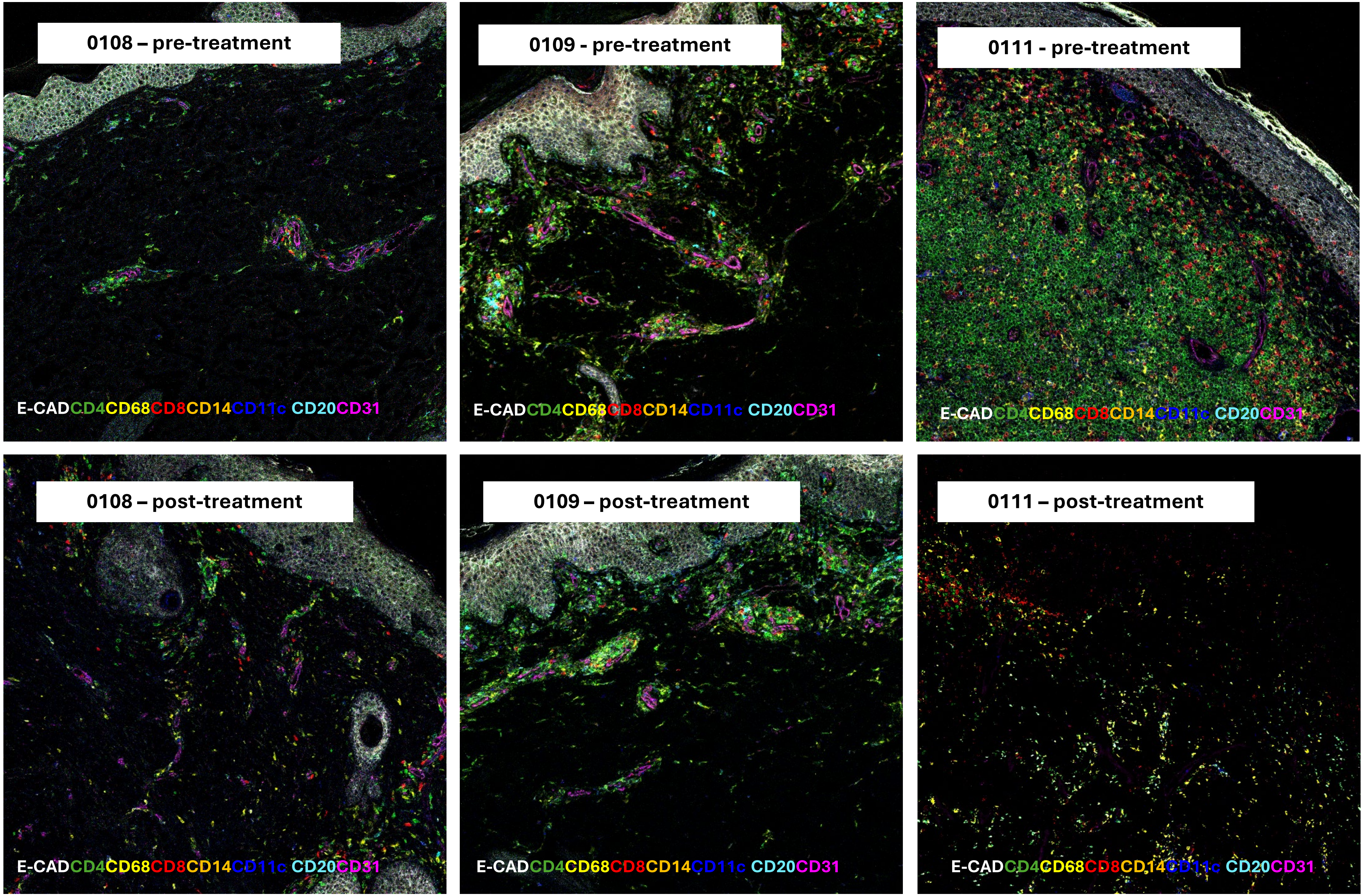

Figure S5. (continued)

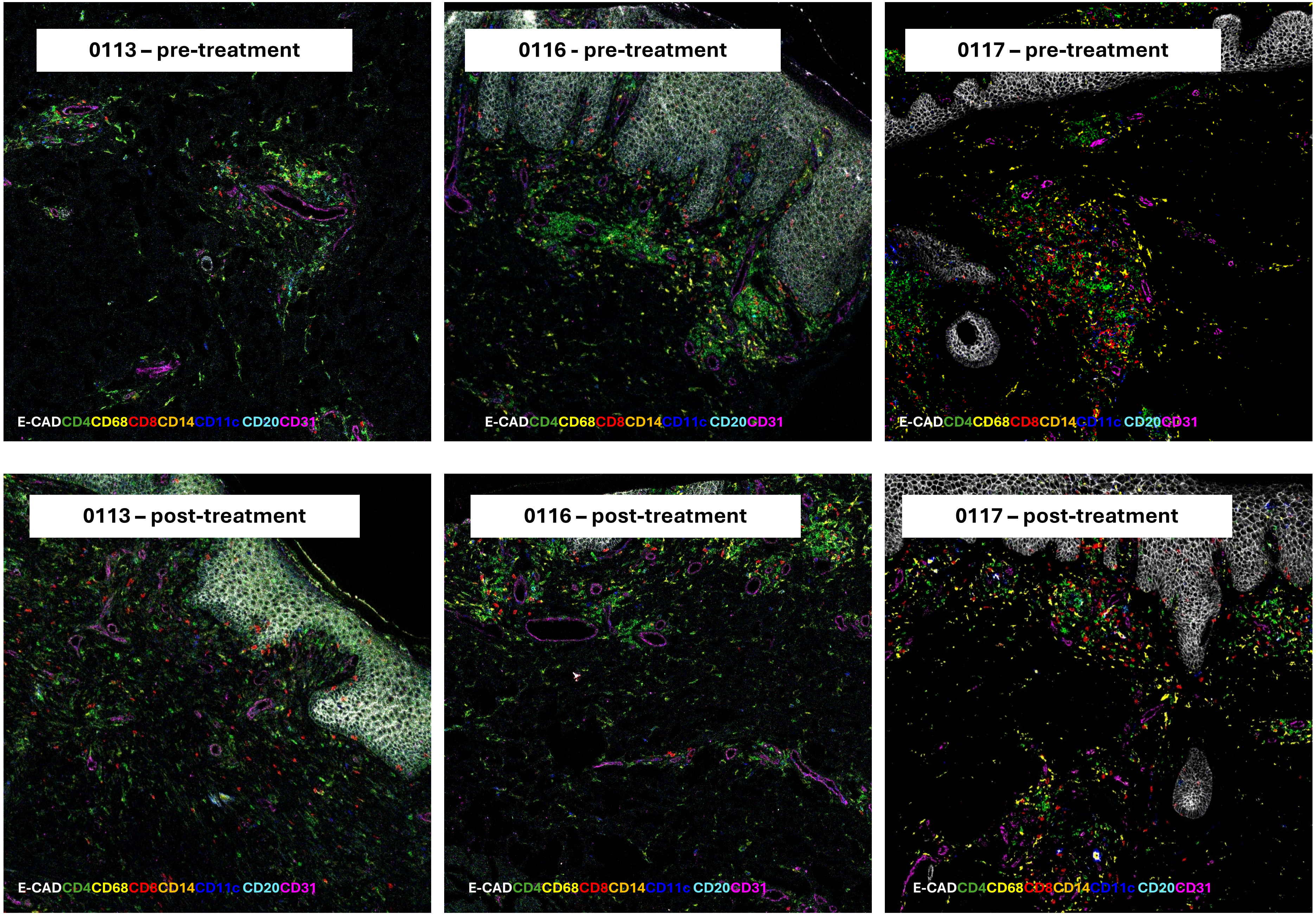

Figure S5.
(continued)

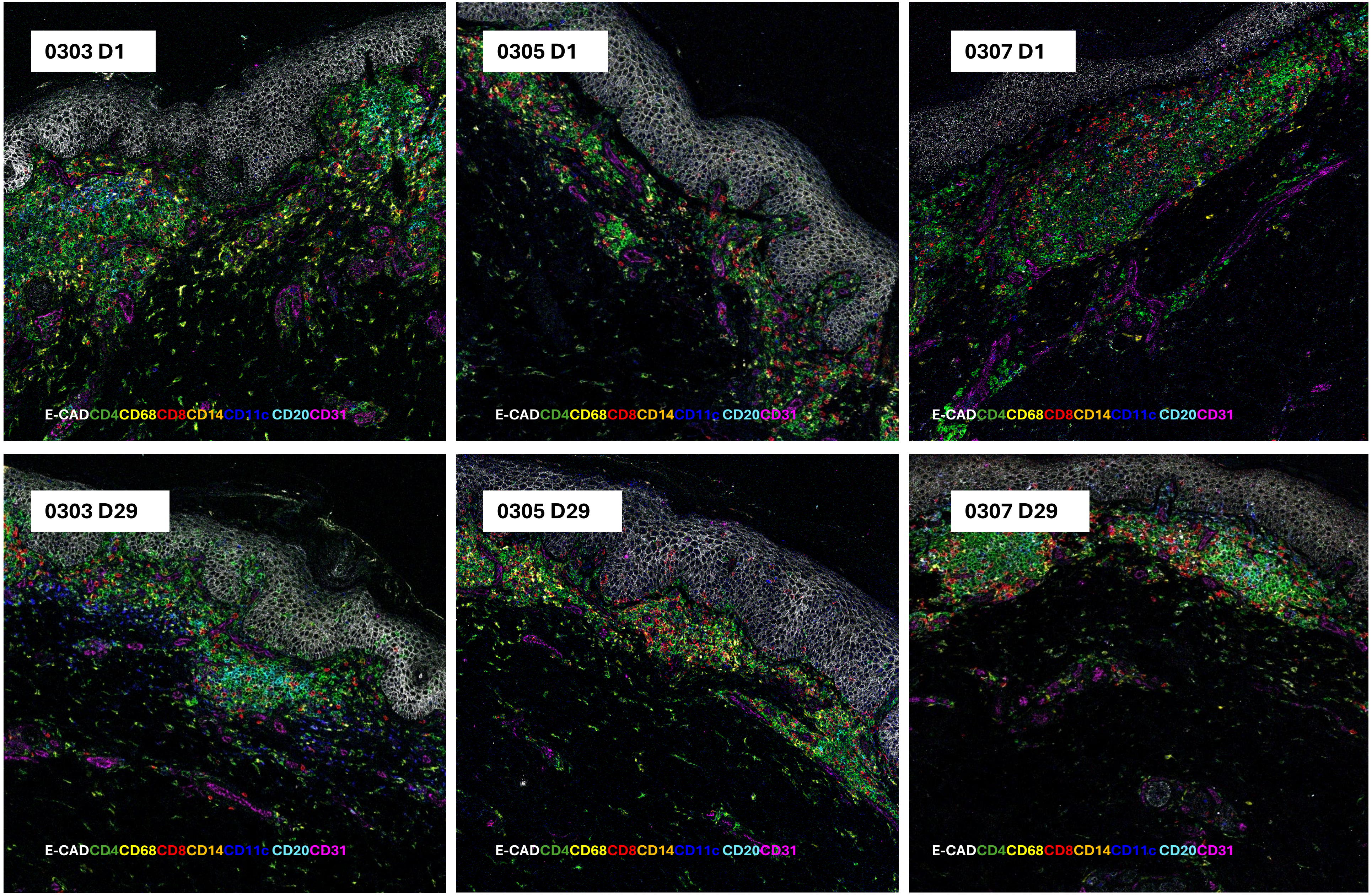

Figure S5.
(continued)

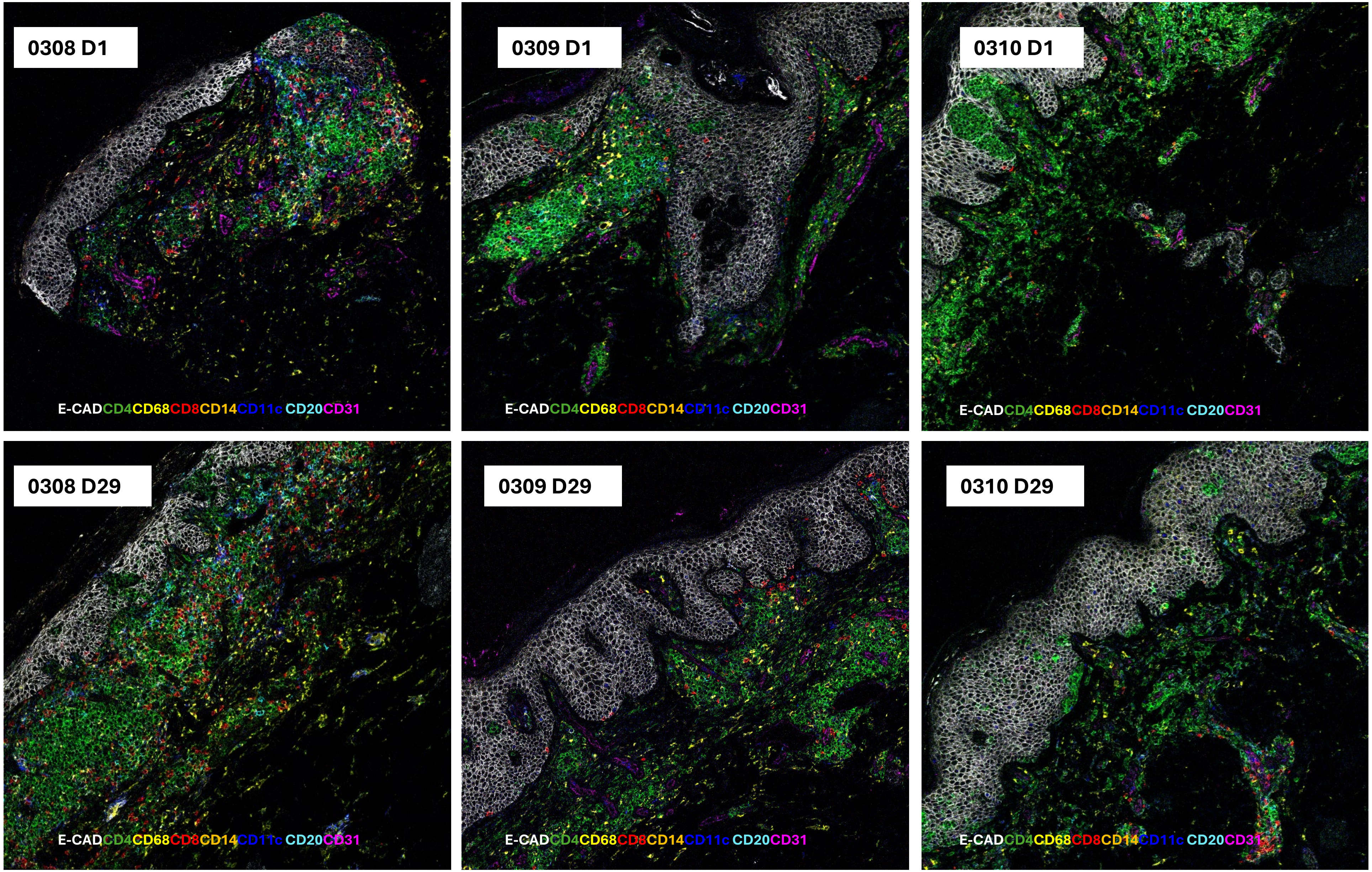

Figure S5. (continued)

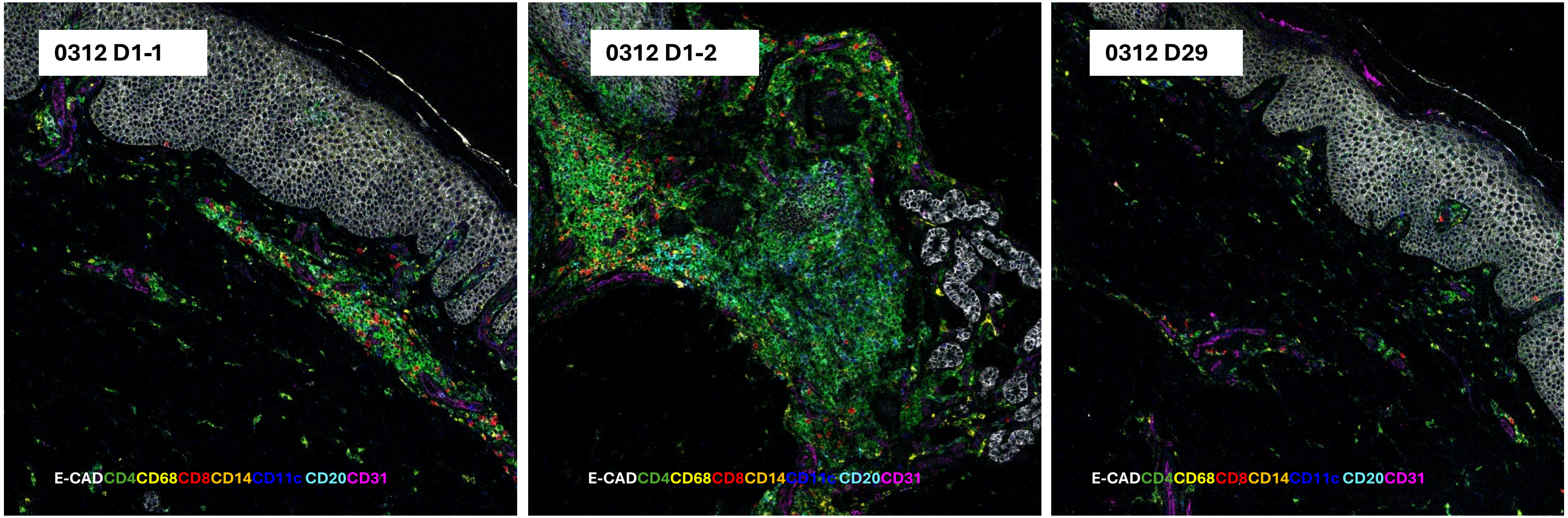

Figure S5.
(continued)

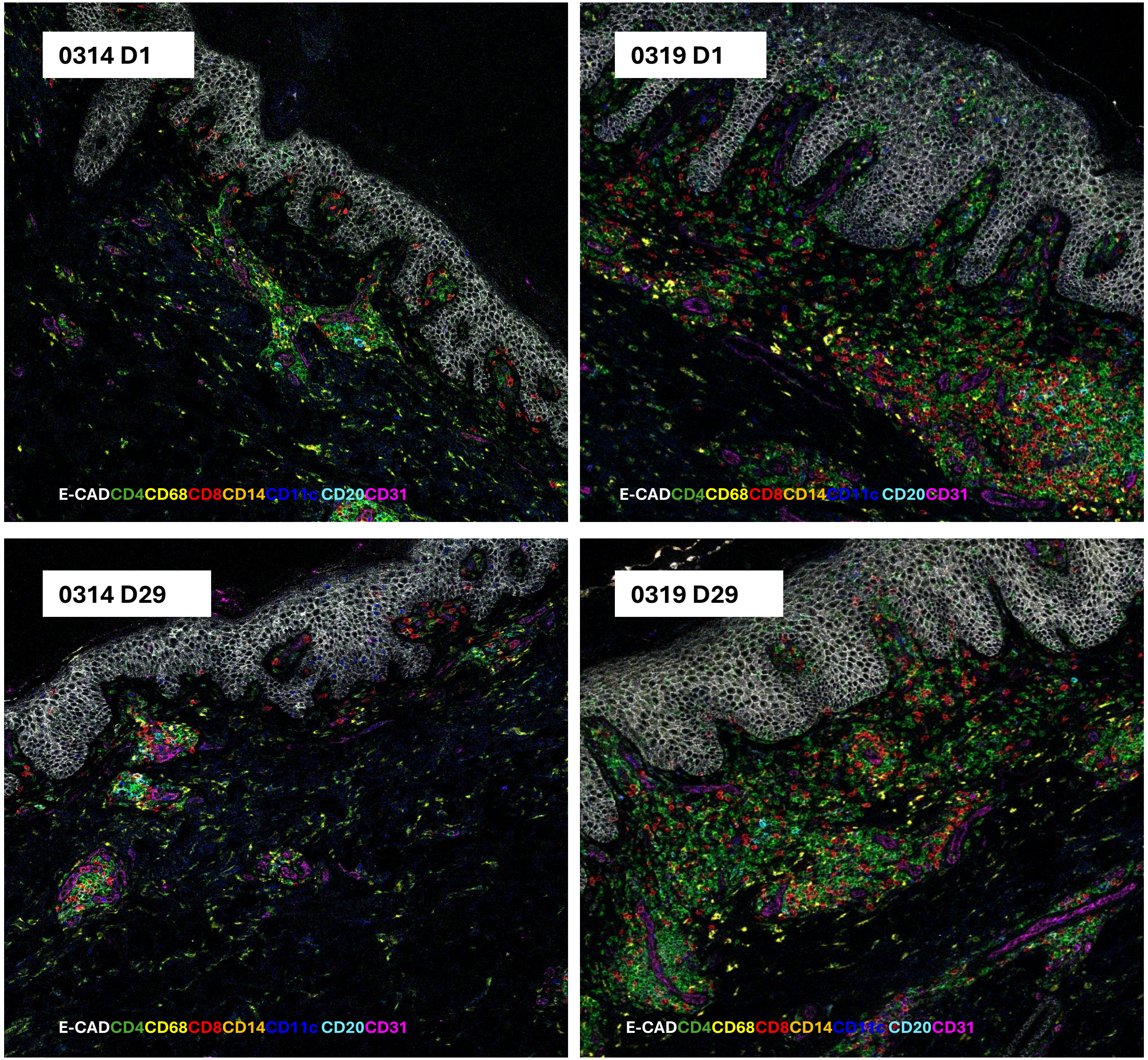

Figure S6. Cell clustering and marker-specific expression identified by unsupervised IMC image analysis with differential abundance between pre- and post-treatment samples

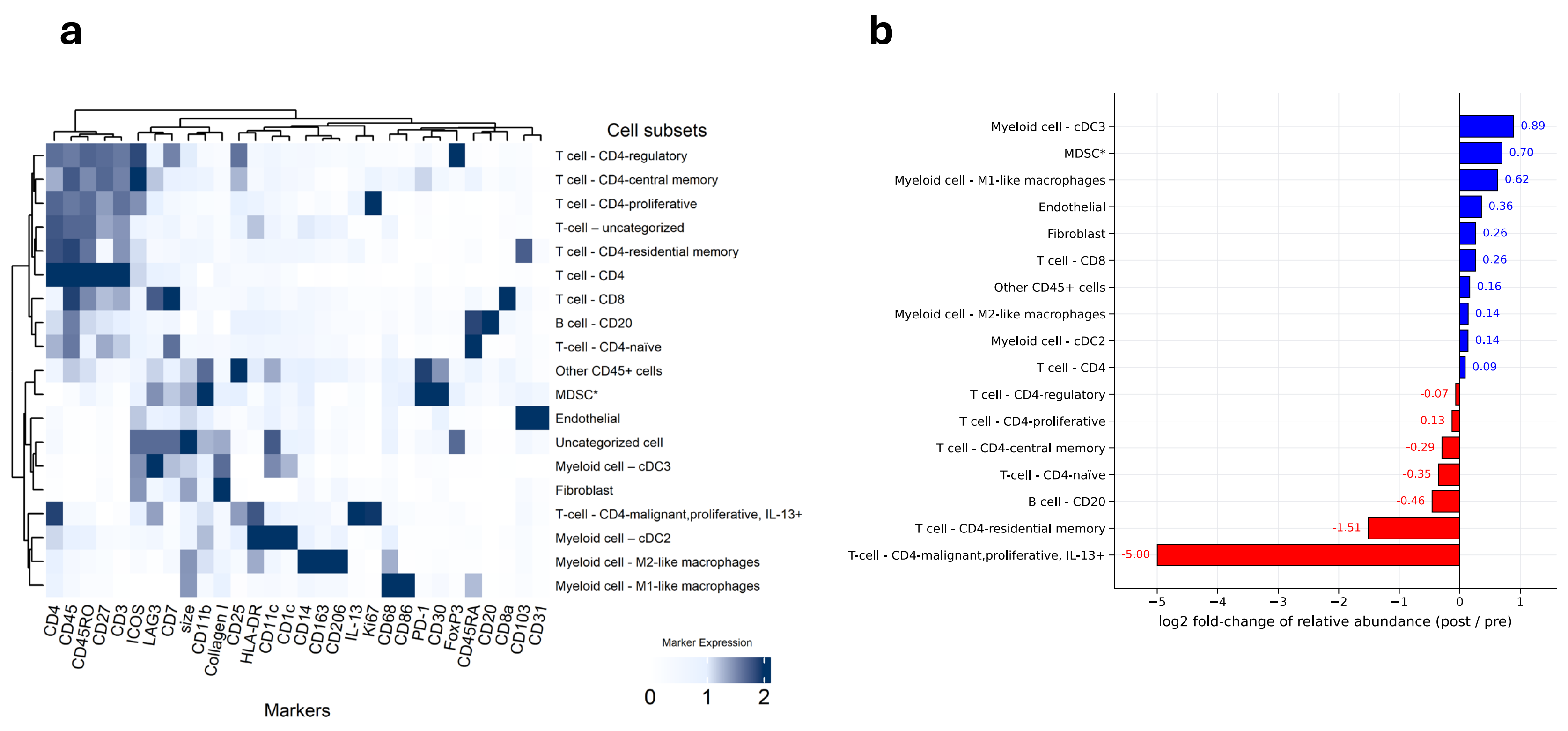

Supplement: Supplementary file 1 [file cancers-18-02348-s001.zip › cancers-4346468-supplementary.pdf]
